# Integrative genomic profiling of large-cell neuroendocrine carcinomas reveals distinct subtypes of high-grade neuroendocrine lung tumors

**DOI:** 10.1038/s41467-018-03099-x

**Published:** 2018-03-13

**Authors:** Julie George, Vonn Walter, Martin Peifer, Ludmil B. Alexandrov, Danila Seidel, Frauke Leenders, Lukas Maas, Christian Müller, Ilona Dahmen, Tiffany M. Delhomme, Maude Ardin, Noemie Leblay, Graham Byrnes, Ruping Sun, Aurélien De Reynies, Anne McLeer-Florin, Graziella Bosco, Florian Malchers, Roopika Menon, Janine Altmüller, Christian Becker, Peter Nürnberg, Viktor Achter, Ulrich Lang, Peter M. Schneider, Magdalena Bogus, Matthew G. Soloway, Matthew D. Wilkerson, Yupeng Cun, James D. McKay, Denis Moro-Sibilot, Christian G. Brambilla, Sylvie Lantuejoul, Nicolas Lemaitre, Alex Soltermann, Walter Weder, Verena Tischler, Odd Terje Brustugun, Marius Lund-Iversen, Åslaug Helland, Steinar Solberg, Sascha Ansén, Gavin Wright, Benjamin Solomon, Luca Roz, Ugo Pastorino, Iver Petersen, Joachim H. Clement, Jörg Sänger, Jürgen Wolf, Martin Vingron, Thomas Zander, Sven Perner, William D. Travis, Stefan A. Haas, Magali Olivier, Matthieu Foll, Reinhard Büttner, David Neil Hayes, Elisabeth Brambilla, Lynnette Fernandez-Cuesta, Roman K. Thomas

**Affiliations:** 1 Department of Translational Genomics, Center of Integrated Oncology Cologne–Bonn, Medical Faculty, University of Cologne, Cologne, 50931 Germany; 2 UNC Lineberger Comprehensive Cancer Center School of Medicine, University of North Carolina at Chapel Hill, Chapel Hill, NC 27599-7295 USA; 3 Department of Biochemistry and Molecular Biology, Penn State Milton S. Hershey Medical Center, 500 University Drive, Hershey, PA 17033 USA; 4 Center for Molecular Medicine Cologne (CMMC), University of Cologne, 50931 Cologne, Germany; 5 Department of Cellular and Molecular Medicine and Department of Bioengineering and Moores Cancer Center, University of California, La Jolla, San Diego, CA 92093 USA; 6 Genetic Cancer Susceptibility Group, Section of Genetics, International Agency for Research on Cancer (IARC-WHO), Lyon, 69008 France; 7 Molecular Mechanisms and Biomarkers Group, Section of Mechanisms of Carcinogenesis, International Agency for Research on Cancer (IARC-WHO), 69008 Lyon, France; 8 Section of Environment and Radiation, International Agency for Research on Cancer (IARC-WHO), 69008 Lyon, France; 9 Computational Molecular Biology Group, Max Planck Institute for Molecular Genetics, 14195 Berlin, Germany; 10 Programme Cartes d’Identité des Tumeurs (CIT), Ligue Nationale Contre le Cancer, 14 rue Corvisart, Paris, 75013 France; 11 CHU Grenoble Alpes, UGA/INSERM U1209/CNRS, Grenoble, France; 12 NEO New Oncology GmbH, 51105 Cologne, Germany; 13 Cologne Center for Genomics (CCG), University of Cologne, 50931 Cologne, Germany; 14 Institute of Human Genetics, University Hospital Cologne, 50931 Cologne, Germany; 15 Cologne Excellence Cluster on Cellular Stress Responses in Aging-Associated Diseases (CECAD), University of Cologne, 50931 Cologne, Germany; 16 Computing Center, University of Cologne, 50931 Cologne, Germany; 17 Department of Informatics, University of Cologne, 50931 Cologne, Germany; 18 Institute of Legal Medicine, University Hospital Cologne, 50823 Cologne, Germany; 19 Department of Genetics, Lineberger Comprehensive Cancer Center, The University of North Carolina at Chapel Hill, NC, 27599-7295 USA; 20 CHUGA Grenoble, INSERM U 1209, University Grenoble Alpes, Institute of Advanced Biosciences (IAB), 38043, CS10217 Grenoble, France; 21 Department of Pathology, CHUGA, INSERM U 1209, University of Grenobles Alpes, Institute of Advanced Biosciences (IAB), 38043, CS10217 Grenoble, France; 22 Department of Biopathology, Centre Léon Bérard UNICANCER, 69008 Lyon, France; 23 Institute of Pathology and Molecular Pathology, University Hospital Zurich, 8091 Zurich, Switzerland; 24 Department of Thoracic Surgery, University Hospital Zurich, 8091 Zurich, Switzerland; 25 Institute of Clinical Medicine, Faculty of Medicine, University of Oslo, N-0424 Oslo, Norway; 26 Department of Oncology, Norwegian Radium Hospital, Oslo University Hospital, N-0310 Oslo, Norway; 27 Department of Pathology, Norwegian Radium Hospital, Oslo University Hospital, N-0310 Oslo, Norway; 28 Department of Thoracic Surgery, Rikshospitalet, Oslo University Hospital, N-0027 Oslo, Norway; 29 Department of Internal Medicine, Center of Integrated Oncology Cologne-Bonn, University Hospital Cologne, 50937 Cologne, Germany; 30 Department of Surgery, St. Vincent’s Hospital, Peter MacCallum Cancer Centre, 3065 Melbourne, Victoria Australia; 31 Department of Haematology and Medical Oncology, Peter MacCallum Cancer Centre, 3065 Melbourne, Victoria Australia; 32 Tumor Genomics Unit, Department of Experimental Oncology and Molecular Medicine, Fondazione IRCCS—Istituto Nazionale Tumori, Via Venezian 1, 20133 Milan, Italy; 33 Thoracic Surgery Unit, Department of Surgery, Fondazione IRCCS Istituto Nazionale Tumori, 20133 Milan, Italy; 34 Institute of Pathology, Jena University Hospital, Friedrich-Schiller-University, 07743 Jena, Germany; 35 Department of Internal Medicine II, Jena University Hospital, Friedrich-Schiller-University, 07743 Jena, Germany; 36 Institute for Pathology Bad Berka, 99438 Bad Berka, Germany; 37 Gastrointestinal Cancer Group Cologne, Center of Integrated Oncology Cologne–Bonn, Department I for Internal Medicine, University Hospital of Cologne, 50823 Cologne, Germany; 38 Department of Pathology, University Hospital Cologne, 50937 Cologne, Germany; 39 Pathology of the University Medical Center Schleswig-Holstein, Campus Luebeck and the Research Center Borstel, Leibniz Center for Medicine and Biosciences, 23538 Luebeck and 23845 Borstel, Borstel, Germany; 40 Department of Pathology, Memorial Sloan-Kettering Cancer Center, New York, NY 10065 USA; 41 German Cancer Research Center, German Cancer Consortium (DKTK), 69120 Heidelberg, Germany

## Abstract

Pulmonary large-cell neuroendocrine carcinomas (LCNECs) have similarities with other lung cancers, but their precise relationship has remained unclear. Here we perform a comprehensive genomic (*n* = 60) and transcriptomic (*n* = 69) analysis of 75 LCNECs and identify two molecular subgroups: “type I LCNECs” with bi-allelic *TP53* and *STK11*/*KEAP1* alterations (37%), and “type II LCNECs” enriched for bi-allelic inactivation of *TP53* and *RB1* (42%). Despite sharing genomic alterations with adenocarcinomas and squamous cell carcinomas, no transcriptional relationship was found; instead LCNECs form distinct transcriptional subgroups with closest similarity to SCLC. While type I LCNECs and SCLCs exhibit a neuroendocrine profile with *ASCL1*^high^/*DLL3*^high^/*NOTCH*^low^, type II LCNECs bear *TP53* and *RB1* alterations and differ from most SCLC tumors with reduced neuroendocrine markers, a pattern of *ASCL1*^low^/*DLL3*^low^/*NOTCH*^high^, and an upregulation of immune-related pathways. In conclusion, LCNECs comprise two molecularly defined subgroups, and distinguishing them from SCLC may allow stratified targeted treatment of high-grade neuroendocrine lung tumors.

## Abstract (web summary)

The molecular nature of large-cell neuroendocrine lung carcinomas (LCNEC) has remained unclear. Here, the authors show LCNECs represent a distinct transcriptional subgroup among lung cancers and comprise two molecular subgroups, type I (TP53 and STK11/KEAP1 alterations) and type II (TP53 and RB1 inactivation).

## Introduction

Molecular characterization studies have provided invaluable insight into the relationship between the major lung tumor subtypes^1–7^. These studies showed that morphologically defined lung adenocarcinomas, squamous cell carcinomas, and small cell carcinomas have distinct molecular phenotypes based upon their somatically altered genes^7^. Furthermore, global transcriptional analyses have revealed intra-group consistency, as well as substantial differences in the patterns of expressed genes, which led to the discovery of novel intra-group subtypes^2,3,8–11^ and to the elimination of previous lung tumor categories (e.g., large-cell carcinoma)^7^. Of the remaining lung cancer subtypes, only large-cell neuroendocrine carcinomas (LCNECs) have so far not been characterized in depth using both transcriptomic, as well as genomic approaches.

LCNECs account for 2–3% of all resected lung cancers and belong to the category of neuroendocrine lung tumors, which also includes pulmonary carcinoids (PCa) and small cell lung cancer (SCLC)^12,13^. Contrary to pulmonary carcinoids, LCNEC and SCLC are clinically aggressive tumors presenting in elderly heavy-smokers with 5-year survival rates below 15–25% (LCNEC) and 5% (SCLC), respectively^12,13^. While therapy for both typical and atypical carcinoids and SCLC is primarily surgery and chemotherapy (in the case of SCLC), chemotherapy has limited efficacy in LCNEC patients and no standard treatment regimen exists for this tumor type^14^. Thus, LCNECs share both commonalities (e.g., neuroendocrine differentiation) and discrepancies (e.g., limited response to chemotherapy) with SCLC; however, the underlying molecular basis of these shared and distinct features is only poorly understood. Further complicating the histological classification, LCNECs are sometimes found combined with adenocarcinoma or squamous cell carcinoma and some SCLCs are combined with a component of LCNEC^12,13^. Thus, defining the molecular patterns of this tumor type presents the opportunity to not only reveal possible novel therapeutic targets, but also help clarifying the ontogeny and relationship of lung tumors in general.

Previous efforts in characterizing LCNECs through targeted sequencing of selected cancer-related genes^15–17^ and through gene expression profiling^18^ provided some first insights; however, global genomic studies combined with transcriptomic analyses have so far been lacking. Furthermore, given the lack of adequate therapeutic strategies in LCNECs, a precise delineation of the molecular boundaries between different neuroendocrine tumors is needed. We therefore aimed to comprehensively dissect both the mutational and the transcriptional patterns of this tumor type.

In this report, we show that LCNECs are composed of two mutually exclusive subgroups, which we categorize as “type I LCNECs” (with *STK11*/*KEAP1* alterations) and “type II LCNECs” (with *RB1* alterations). Despite sharing genomic alterations with lung adenocarcinomas and squamous cell carcinomas, type I LCNECs exhibit a neuroendocrine profile with closest similarity to SCLC tumors. While type II LCNECs reveal genetic resemblance to SCLC, these tumors are markedly different from SCLC with reduced levels of neuroendocrine markers and high activity of the *NOTCH* pathway. Conclusively, LCNECs represent a distinct subgroup within the spectrum of high-grade neuroendocrine tumors of the lung, and our findings emphasize the importance of distinguishing LCNECs from other lung cancers subtypes.

## Results

### Genomic alterations in LCNECs

We collected 75 fresh-frozen tumor specimens from patients diagnosed with LCNEC under institutional review board approval (Supplementary Data 1). All tumors were thoroughly analyzed, and the histological features of pulmonary LCNECs were confirmed by expert pathologists (E.B., W.T., R.B.) according to the 2015 WHO classification^13^ (Supplementary Data 2). Most tumors were obtained from current or former heavy smokers, and enriched for stages I and II (68%). Nineteen of 75 LCNECs included in this study showed additional histological components of lung adenocarcinoma (ADC) (*n* = 2), squamous cell carcinoma (SqCC) (*n* = 5) or SCLC (*n* = 12) (Supplementary Data 1–2). In subsequent analyses nucleic acids were extracted only from pure LCNEC regions (Methods section).

**Fig. 1:**
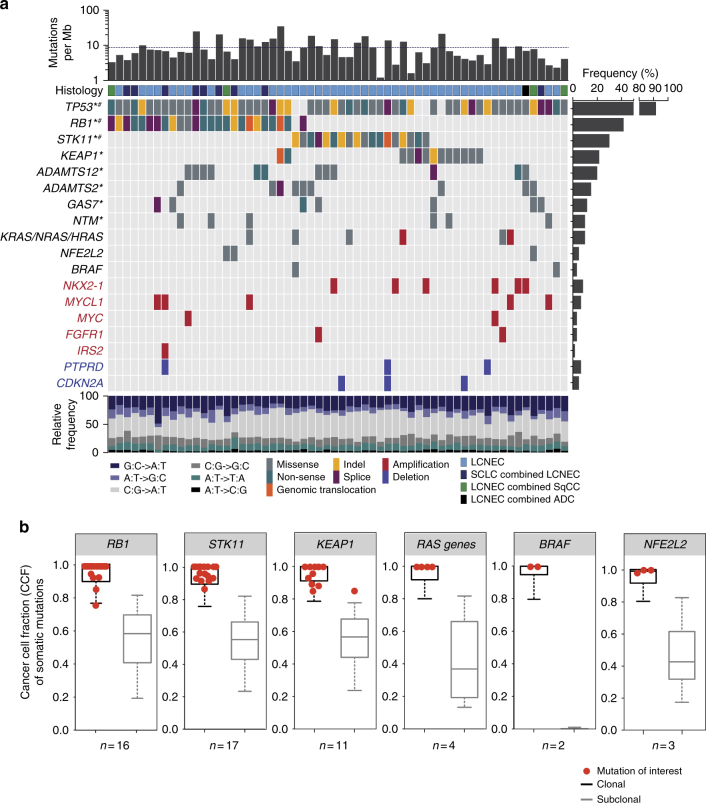
Genomic alterations in pulmonary large-cell neuroendocrine carcinomas (LCNECs). **a** Tumor samples are arranged from left to right. Histological assignments and somatic alterations in candidate genes are annotated for each sample according to the color panel below the image. The somatic mutation frequencies for each candidate gene are plotted on the right panel. Mutation rates and the type of base-pair substitutions are displayed in the top and bottom panel, respectively; a dashed black line indicates the average value. Significantly mutated genes and genes with a significant enrichment of damaging mutations are denoted with * and #, respectively (*Q* < 0.01, Methods section). Genes with significant copy number (CN) amplifications (CN > 4) and deletions (CN < 1) (Supplementary Fig. 2a, Supplementary Dataset 5) are displayed in red and blue, respectively (*Q* < 0.01, Methods section). **b** The distribution of clonal and sub-clonal mutations was analyzed for tumor samples that harbored mutations in key candidate genes. The cancer cell fractions (CCF) of all mutations were determined, assigned to clonal or sub-clonal fractions (Methods section), and displayed as whiskers box-plot (median and interquartile range, whiskers: 5–95 percentile). The CCF of candidate gene mutations is highlighted in red

Early genomic profiling studies employing targeted sequencing of selected cancer-related genes aided in the identification of some prevalent mutations in LCNECs^15–17^. In order to assess globally all genomic alterations in LCNECs and to compare them to those occurring in other lung tumors, we conducted whole-exome sequencing (WES) of 55 LCNEC tumor-normal pairs; we additionally performed whole-genome sequencing (WGS) in those cases where sufficient material was available (*n* = 11), thus amounting to sequencing data of 60 LCNECs in total (six tumors were both, genome- and exome-sequenced, Supplementary Fig. 1a). We furthermore performed Affymetrix 6.0 SNP array analyses of 60 and transcriptome sequencing of 69 tumors (Supplementary Data 1; Supplementary Fig. 1a). Despite initial review to include cases with a microscopic tumor content of >70%, sequencing data analysis revealed a median tumor purity of 59.5% and a median ploidy of 2.8 (Supplementary Data 1, Supplementary Fig. 1b, Methods section). On average, LCNECs exhibited an exonic mutation rate of 8.6 non-synonymous mutations per million base pairs and a C:G > A:T transversion rate of 38.7% (Fig. 1a, Supplementary Data 1), indicative of tobacco exposure^1–6^. We analyzed the signatures of mutational processes^19,20^ in LCNECs, which confirmed a prominent smoking-related signature (signature 4^19,20^) that accounts for the majority of all somatic mutations, and which is in general comparable to most other lung tumors of heavy smokers (Supplementary Fig. 1c–f, Supplementary Data 3).

Analyses of chromosomal gene copy numbers revealed statistically significant amplifications of 1p34 (containing the *MYCL1* gene, 12%), 8p12 (containing *FGFR1*, 7%), 8q24.21 (containing *MYC*, 5%), 13q33 (containing *IRS2*, 3%), and 14q13 (containing *NKX2-1*, also known as TTF-1, 10%) (*Q* < 0.01, Supplementary Fig. 2a; Supplementary Data 4–5, Methods section). Statistically significant deletions affected *CDKN2A* (9p21, 8%) and a putative fragile site at *PTPRD* (9p24, 7%)^21^. While amplifications of *NKX2-1* and *FGFR1* frequently occur in lung adenocarcinomas^1,2,7,21^ and squamous cell carcinomas^3,7,21,22^, respectively, *MYCL1* amplifications are commonly found in SCLC^4–6,23^. Thus, LCNECs harbor significant copy-number alterations that occur in different lung cancer subtypes.

We next applied analytical filters to identify mutations with biological relevance in the context of a high-mutation rate and found eight significantly mutated genes (*Q* < 0.01, Methods section, Fig. 1a, Supplementary Data 6–7). *TP53* was the most frequently mutated gene (92%), followed by inactivating somatic events in *RB1* (42%); bi-allelic alterations in both genes, *TP53* and *RB1*—a hallmark of SCLC^4–6^—were found in 40% of the cases (Supplementary Fig. 2b, Supplementary Data 6–9). Notably, LCNECs with admixtures of other histological components mostly had *RB1* alterations (Fig. 1a). While genomic alterations in *RB1* resulted in loss-of-nuclear Rb1 expression (*P* < 0.0001, Fisher’s exact test, Supplementary Fig. 3a), immunohistochemistry revealed that absence of Rb1 was not only confined to the LCNEC component, but also evident in the combined other histological subtype (6/7 cases, Supplementary Fig. 3b, Supplementary Data 2). This may implicate shared genetic features between LCNECs and the admixtures of other histological carcinoma types.

We furthermore identified—frequently deleterious—somatic alterations in functionally relevant domains of *STK11* (30%) and *KEAP1* (22%)^1–3^ (Fig. 1a, Supplementary Fig. 4a, Supplementary Data 6–9). Combined with loss-of-heterozygosity (LOH), bi-allelic alterations of *STK11* and *KEAP1* were found in 37% of the cases (Supplementary Fig. 2b, Supplementary Data 8). In those cases where WGS  was performed, we were able to identify larger genomic rearrangements, which led to the inactivation of *RB1*, *STK11*, or *KEAP1* (Fig. 1a, Supplementary Fig. 4a, Supplementary Data 9). Altogether, somatic alterations of *RB1* and *STK11*/*KEAP1* were detected in 82% of the cases (*n* = 49) and occurred in a mutually exclusive fashion (*P* < 0.0001, Fisher’s exact test, Fig. 1a). We furthermore observed a trend toward inferior outcome in patients with *RB1*-mutated tumors (*P* = 0.126, log-rank test, Supplementary Fig. 4b). The genomic profiling thus points to two distinct subgroups of LCNECs.

We additionally identified statistically significant mutations in the metalloproteinases *ADAMTS2* (15%) and *ADAMTS12* (20%), and in *GAS7* (12%) and *NTM* (10%) (*Q* < 0.01, Methods section, Fig. 1a, Supplementary Fig. 4c, Supplementary Data 6–7), which so far have not been reported as significantly mutated in any other lung cancer subtype. The mutations affected functionally important protein domains, which may suggest a relevant role in the tumorigenesis of LCNECs (Supplementary Fig. 3c).

We also analyzed LCNECs for alterations in genes of known tumor-specific functions (e.g., *CREBBP*, *EP300*^3,4,6,21^, *NOTCH*^3,6,21^, *MEN1*^24^, *ARID1A*^1–3,21,24^) (Supplementary Fig. 2b, Supplementary Fig. 4d, Supplementary Data 6) and found oncogenic mutations of *RAS* family genes (*KRAS*-G12V, -G12C, *NRAS*-D57E, *HRAS*-G13R), *NFE2L2* (2 cases with G31V and 1 case with E79Q) and *BRAF* (V600E, and G469V). Combined with focal amplifications, *RAS* genes were affected in 10% of the tumors (Fig. 1a; Supplementary Data 5–6). We also identified several private in-frame fusion events, e.g., involving the kinases *NTRK1* and *PTK6*, which were, however, not recurrent (Supplementary Fig. 5, Supplementary Data 10). Thus, LCNECs harbor alterations of oncogenes which are commonly found in lung adenocarcinomas, but usually absent in neuroendocrine tumors like SCLC.

The distinct mutational patterns in LCNECs and the presence of other histological components may suggest a high level of intra-tumor heterogeneity. We analyzed the clonal distribution of somatic alterations and determined the cancer cell fraction (CCF) of each somatic mutation call (Methods section). Despite the fact that some LCNECs were found with admixtures of other histological subtypes (Fig. 1a, Supplementary Data 1–2), our studies on the LCNEC component of such composite tumors pointed to little intra-tumor heterogeneity with a median of 7% sub-clonal mutations per sample (Supplementary Fig. 2b–c, Supplementary Data 1, Methods section). Furthermore, all relevant and significant mutations were found to be clonal within the tumor, thus suggesting these alterations as early events during tumorigenesis (Fig. 1b, Supplementary Data 6).

In summary, genome sequencing revealed distinct genomic profiles in LCNECs. While certain alterations (e.g., *RB1, MYCL1*) resemble patterns found in SCLC^4–6,23^, others are typical of lung adenocarcinoma or squamous cell carcinomas (e.g., *STK11*, *KEAP1*, *NKX2-1*, *RAS*, *BRAF*, and *NFE2L2*)^1–3,7,21^. Thus, LCNECs appear to divide into molecularly defined subsets of tumors with genomic similarities to other major lung cancer subtypes.

### Transcriptional profiles of LCNECs and other lung cancers

Our sequencing efforts have revealed genomic alterations in LCNECs that were previously known as canonical alterations in either, lung adenocarcinomas, squamous cell carcinomas^7,21^, or SCLC^4–6^. In light of these distinct associations, it remained to be understood if these genomic correlates might reflect a relationship of LCNECs with these lung tumor subtypes on the level of gene expression. We therefore analyzed whether the transcriptional patterns in LCNECs are correlated with the expression profiles of other lung cancers.

**Fig. 2:**
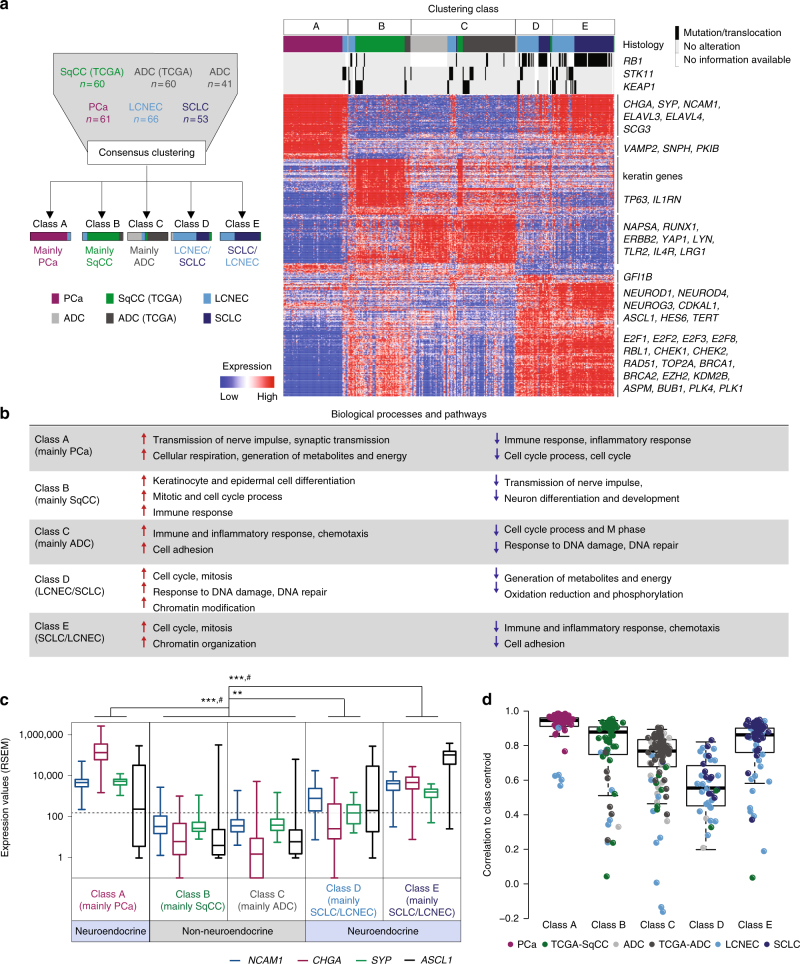
Gene expression studies on lung cancer subtypes. **a** A schematic description of the unsupervised consensus clustering approach is provided on the left panel. The clustering results are displayed on the right panel as a heatmap, in which tumor samples are arranged in columns, grouped according to their expression clustering class, annotated for the histological subtype and for the somatic alteration status. Expression values of genes identified by ClaNC (Methods section) are represented as a heatmap; red and blue indicate high and low expression, respectively. Selected candidate genes are shown on the right. **b** Significant enrichment of differentially expressed genes in signaling pathways is displayed for all clustering classes (*P* < 0.0001, Methods section). **c** Expression values for key neuroendocrine differentiation markers are plotted for each clustering class as box-plots (median and interquartile range, whiskers: min–max values). Dashed black lines indicate the threshold for low expression (Methods section). *Q* < 0.05 (#), significance determined by SAM (Supplementary Dataset 12); *P* < 0.001 (***) Mann–Whitney *U*-test. **d** The correlation of each sample to the centroid of its clustering class was calculated and displayed as box-plot (median and interquartile range, whiskers 5–95 percentile)

We compared the expression data of LCNECs with lung adenocarcinomas^2,3,25–27^, squamous cell carcinomas^3^, SCLC^6^ and pulmonary carcinoids^24^ following extensive normalization of the transcriptome sequencing data (Fig. 2a, Methods section, Supplementary Data 11). Unsupervised consensus clustering yielded five consistent expression clusters, which correlated with the histological annotation of the tumors (*P* < 0.0001, Fig. 2a, Supplementary Fig. 6–7, Supplementary Data 12): pulmonary carcinoids, squamous cell carcinomas and adenocarcinomas formed distinct transcriptional classes (classes A, B, and C, respectively), with few LCNECs falling into these groups. However, the majority of SCLC and LCNECs clustered in two transcriptional subgroups (classes D and E) (Fig. 2a); a phenomenon that had previously been observed in other studies on high-grade neuroendocrine tumors^6,18^. While the majority of SCLC tumors formed consensus cluster E (75% of all SCLC cases analyzed), a fraction of SCLC tumors shared transcriptional similarities with LCNECs that predominantly formed cluster D. Thus, LCNECs appear to be more closely related to SCLCs than to adenocarcinomas or squamous cell carcinomas.

We next analyzed the transcriptome sequencing data for differentially expressed genes and their enrichment in biological pathways (Methods section). In line with previous observations^2,3,9–11,18,28^, this analysis showed that both adenocarcinomas and squamous cell carcinomas exhibited upregulation of pathways controlling cell differentiation, adhesion and immune responses, along with higher expression of *ERBB2* and *TP63* (Fig. 2b, Supplementary Fig. 8a, Supplementary Data 13–14, *Q* < 0.05, Methods section). Lung neuroendocrine tumors, on the contrary, showed significantly higher expression of neuroendocrine and endocrine markers, Hu antigens (*ELAVL3* and *ELAVL4*) and the lineage transcription factor and oncogene *ASCL1*, which is in agreement with previous studies on lung cancer subtypes^11–13,18,29^ (*Q* < 0.05, Methods section). Furthermore, particularly high expression of the neuronal and endocrine lineage transcription factors *NEUROD1*, *NEUROD4*, and *NEUROG3*^30,31^ was found in SCLC and LCNECs of transcriptional class E (Fig. 2a, c, Supplementary Fig. 8b–e, Supplementary Data 13, *Q* < 0.05). While recent studies employing SCLC cell lines and mouse models indicated discordant expression patterns for *ASCL1* and *NEUROD1*^31^, our sequencing data of human high-grade neuroendocrine lung tumors revealed expression of both neuroendocrine lineage factors in class E (Supplementary Fig. 8f).

Within the spectrum of neuroendocrine lung tumors, pulmonary carcinoids formed a distinct subgroup with functional enrichment in pathways regulating cellular respiration and metabolism. LCNECs mostly shared similarities with SCLC, revealing upregulation of pathways and genes controlling cell cycle and mitosis (E2F transcription factors and checkpoint kinases), DNA damage response (*RAD51*, *TOP2A*, and *BRCA1*) and centrosomal functions (such as *BUB1*, *PLK1*, and *ASPM*); which, to some extent, were also found in squamous cell carcinomas (Fig. 2b; Supplementary Fig. 8g–i, Supplementary Data 13–14), and which is in agreement with previous studies^18^. Further supporting a molecular relationship of SCLC and LCNECs in a fraction of the cases, *RB1*-mutated LCNECs were enriched in classes D and E (*P* < 0.05, Fisher’s exact test). Although, LCNECs also harbored alterations commonly observed in adenocarcinomas and squamous cell carcinomas, even LCNECs with such alterations in *KEAP1* or *STK11* were primarily found in transcriptional subclasses shared with SCLC (Fig. 2a, Supplementary Fig. 7c, Supplementary Data 12). Therefore, this observation supports the view that despite the similarity in oncogenic mutations, LCNECs rather constitute their own biological class; and may not be considered as neuroendocrine versions of adenocarcinomas or squamous cell carcinomas.

We also quantified the consistency of the expression profiles for each sample with respect to its clustering group. Again, this analysis revealed a strong correlation for most LCNECs clustering with SCLC tumors (classes D and E); on the other hand, expression profiles of those few LCNEC samples clustering with lung adenocarcinomas, squamous cell carcinomas, and pulmonary carcinoids were less consistent (Fig. 2d). Furthermore, we performed separate transcriptional clustering of LCNECs with adenocarcinomas and squamous cell carcinomas only (excluding SCLC), which did not suggest any unrecognized similarities between these lung cancer subtypes (Supplementary Fig. 9). Thus, despite sharing somatic alterations with other tumor subtypes, such as adenocarcinomas and squamous cell carcinomas, LCNECs were transcriptionally dissimilar with all non-neuroendocrine lung tumors and showed closest similarities to SCLC.

### The transcriptional relationship of LCNEC and SCLC

**Fig. 3:**
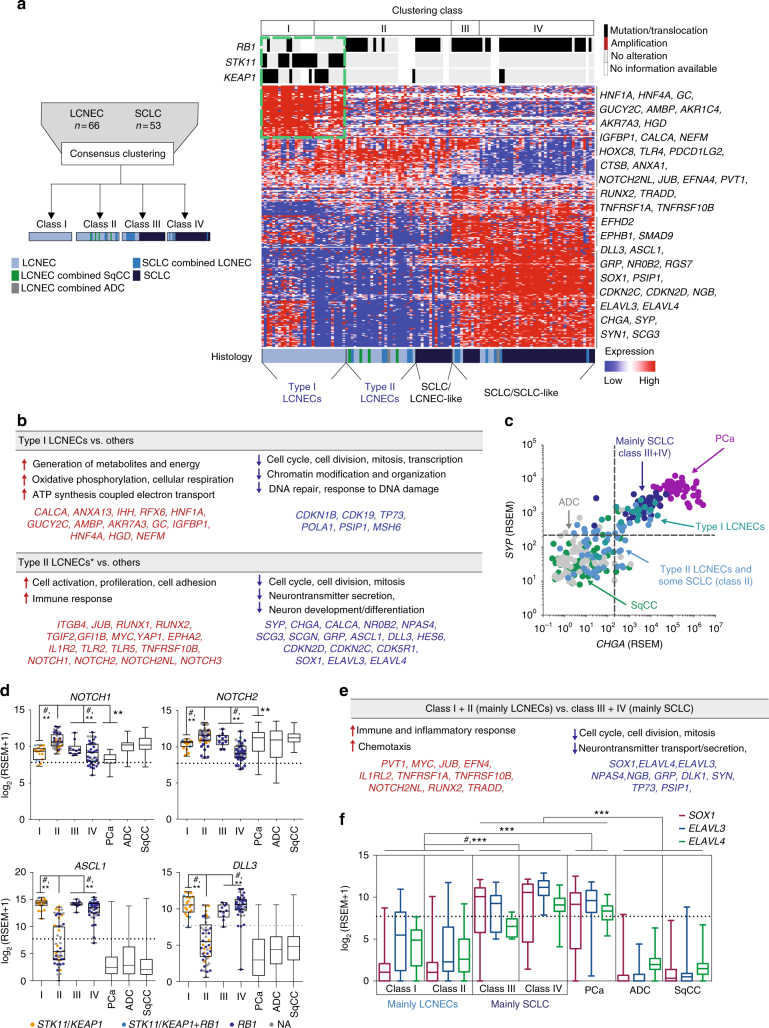
Gene expression studies on LCNEC and SCLC. **a** The expression profiles of LCNEC and SCLC tumors were analyzed following the annotation and approach described in Fig. 2a. Expression values of genes identified by ClaNC (Methods section) are represented as a heatmap in which red and blue indicate high and low expression, respectively.  Selected candidate genes are shown on the right. Dashed green lines indicate an expression profile shared by LCNEC tumors with *STK11*/*KEAP1* alterations (type I LCNECs). **b** The significant enrichment of differentially expressed genes and signaling pathways are displayed for type I LCNECs and type II LCNECs. *P* < 0.0001 (Methods section); * some SCLC tumors that co-clustered with type II LCNECs were included in this analysis. Key candidate genes are highlighted in bold. **c**, **d** Expression values for **c** the key neuroendocrine differentiation markers *SYP* (synaptophysin) and *CHGA* (chromogranin A) (scatter plot), and **d** *NOTCH* pathways genes (box plots: median and interquartile range, whiskers: min–max values). **e** Significant enrichment of differentially expressed genes and signaling pathways was analyzed for class I and II vs class III and IV tumor samples; *P* < 0.0001 (Methods section). **f** Expression values of *SOX1*, *ELAVL3*, and *ELAVL4* are plotted for the clustering classes and other lung cancer subtypes (box plots: median and interquartile range, whiskers: min–max values). *Q* < 0.05 (#), SAM (Supplementary Dataset 12); *P* < 0.01 (**) Mann–Whitney *U*-test

In the previous section, we sought for a global approach to identify common and distinct transcriptional profiles of LCNECs in relationship with other lung tumors, which showed that LCNEC and SCLC appear to share most transcriptional patterns. However, strongly divergent tumors (e.g., carcinoids, adenocarcinomas) may drive these clusters and mask important differences between LCNECs and SCLC. We therefore sought to directly compare LCNECs and SCLC on the transcriptional level (Fig. 3a). The resulting unsupervised clustering analysis revealed four consensus clusters of LCNEC and SCLC that we termed classes I–IV in order to distinguish them from the above-mentioned classes A–E (Fig. 3a, Supplementary Fig. 10–11, Supplementary Data 12). Class I exclusively included LCNECs with *STK11* or *KEAP1* alterations; yet, a few cases with these alterations fell into class II that predominantly consisted of LCNECs with *RB1* loss (Fig. 3a). Some LCNECs, including tumors admixed with SCLC (“SCLC combined LCNECs”)—clustered with the majority of SCLC tumors in the classes III and IV; similarly, some SCLC tumors were part of class II that included LCNECs bearing *RB1* alterations (Fig. 3a, Supplementary Fig. 11). Even though pathological review had been conducted to distinguish histological subtypes from one another, transcriptional clustering suggested high degrees of similarity for some LCNEC and SCLC cases; these tumors may therefore be considered as “SCLC-like” and “LCNEC-like” (Fig. 3a, Supplementary Fig. 11, Supplementary Data 11). Other major genome alterations (e.g., *NKX2-1*, *MYCL1*, *RAS* genes, *NFE2L2*, *BRAF*) did not segregate with the identified transcriptional subgroups (Supplementary Fig. 11). We further analyzed the consistency of the transcriptional subgroups by clustering LCNECs alone, which revealed high concordance with the transcriptional classes identified in Fig. 3a (62/66 cases, 94%, *P* < 0.001, Fisher’s exact test, Supplementary Fig. 13, Supplementary Data 12). Thus, despite the similarities between LCNECs and SCLCs, subtypes of LCNECs exist with profound differences to SCLC.

The transcriptional clustering heatmap pointed to a strong gene expression pattern shared by all LCNECs bearing *STK11*/*KEAP1* alterations (Fig. 3a, Supplementary Fig. 12a, green box in upper left quadrant). We therefore conducted a supervised analysis of the gene expression data, in which LCNECs with *STK11*/*KEAP1* alterations were compared to tumors bearing *RB1* alterations. This analysis indicated specific expression profiles, which were similar to those observed in tumors constituting class I (Fig. 3b, Supplementary Fig. 12, Supplementary Data 13). We therefore assigned this genomic subset of tumors to one group, termed “type I LCNECs”.

Type I LCNECs exhibited high levels of calcitonin A (*CALCA*), a known marker of pulmonary neuroendocrine cells^32–34^ (Fig. 3a, Supplementary Fig. 12b, Supplementary Data 13). This subgroup furthermore displayed a pronounced upregulation of cellular metabolic pathways, which we also observed in pulmonary carcinoids (Fig. 2b), but which was less prominent in LCNECs and SCLC tumors with *RB1* alterations (Fig. 3a, b, Supplementary Data 12–13). Other genes found in type I LCNECs included gastrointestinal transcription factors (e.g., *HNF4A*, *HNF1A*, and *RFX6*), which were previously reported to play a role in de-differentiated lung tumors^35,36^ (Fig. 3b, Supplementary Fig. 12c, d, Supplementary Data 13).

The most striking difference was found in the expression levels of neuroendocrine genes: while type I LCNECs and the majority of SCLC tumors (class III + IV) harbored high levels of neuroendocrine genes (*CHGA* and *SYP*; Fig. 3c; Supplementary Fig. 12e; Supplementary Data 12), most LCNECs and some SCLC tumors with *RB1* alterations in class II exhibited low levels of these genes (Fig. 3c, Supplementary Fig. 12e). By contrast, tumors in class II displayed elevated expression of genes associated with active Notch signaling (e.g., *NOTCH1*, *NOTCH2*, and *HES1*) and immune cell responses (e.g. *PDCD1LG2*, *TLR4*, and *CTSB*) (Fig. 3a, d, Supplementary Fig. 12f, Supplementary Data 12–13). Given the strong enrichment of LCNECs with *STK11* or *KEAP1* alterations in cluster I, and the prominent lack of expression of key neuroendocrine genes in most tumors of class II, we termed LCNECs within this transcriptional class as “type II LCNECs”.

We have recently demonstrated that SCLC tumors usually harbor inactive Notch signaling and that activation of Notch reduces expression of neuroendocrine genes (e.g., *CHGA*, *SYP* and *NCAM1*) and Ascl1^6^. Consistent with this notion, we found that type II LCNECs and some SCLC within this transcriptional class exhibited signs of *NOTCH* upregulation and low expression of neuroendocrine markers, *ASCL1* and *DLL3*, an inhibitor of the Notch signaling pathway^37^ (Fig. 3d, and Supplementary Fig. 12f). Conversely, type I LCNECs and the majority of the SCLC samples (class III and IV) showed higher levels of neuroendocrine genes, as well as of *ASCL1* and *DLL3*, and downregulation of *NOTCH* pathway genes (Fig. 3d, Supplementary Fig. 12f). Thus, despite the fact that type II LCNECs and some SCLCs harbor bi-allelic loss of *TP53* and *RB1*, their transcriptional signatures include low levels of neuroendocrine genes and a distinct profile of *NOTCH*^high^ and *ASCL1*^low^/*DLL3*^low^, which differentiates these tumors from type I LCNECs and from the majority of SCLC cases. We did not identify any significant enrichment of somatic alterations in *NOTCH* pathway genes, which may explain these transcriptional differences (Supplementary Fig. 11). However, a recent study in a pre-clinical mouse model has established a central role of *REST* as a repressor of neuroendocrine markers in SCLC^38^. Compatible with these findings, type II LCNECs displayed significantly higher levels of *REST* (clustering class II, Supplementary Data 12, *Q* < 0.05), which may explain the low neuroendocrine phenotype in type II LCNECs marked by *ASCL1*^low^/*DLL3*^low^/*NOTCH*^high^. Given the important role of NOTCH signaling and *ASCL1* in the decision of neuroendocrine fate and the development of neuroendocrine lung tumors^29,31,38^, these findings provide further support for our distinction of type I and II LCNECs.

We next analyzed the relationship of the expression classes I–IV using hierarchical clustering, which revealed two major subgroups (Supplementary Fig. 11): one subgroup mainly consisting of LCNECs (type I and II LCNECs), and the other subgroup mainly containing SCLC tumors (classes III and IV). Thus, despite harboring distinct transcriptional subcategories, LCNEC and SCLC tumors largely followed their histological annotation and formed separate transcriptional subgroups. Differentially expressed genes included *SOX1* and the neuroendocrine Hu genes (*ELAVL3*, *ELAVL4*), which were enriched in most SCLC samples (classes III and IV (Supplementary Data 13, *Q* < 0.05, Methods section) (Fig. 3f). This observation is in line with previous reports on auto-antibodies against Sox1 and Hu-proteins that are commonly found in SCLC patients^39^. While pulmonary carcinoids harbored similar expression levels, these genes were essentially absent or only moderately expressed in most LCNECs and other lung cancer subtypes (Fig. 3f).

We furthermore analyzed the impact of transcriptional subgroups on tumor stage and clinical outcome. While, we found no association of tumor stage with the molecular subsets found in high-grade neuroendocrine tumors (Supplementary Data 12), we observed a trend toward inferior survival in patients with SCLC (transcriptional profiles of classes III and IV; *P* = 0.072, log-rank test, Supplementary Fig. 14), which was similarly observed in previous studies on high-grade neuroendocrine lung tumors^18^.

**Fig. 4:**
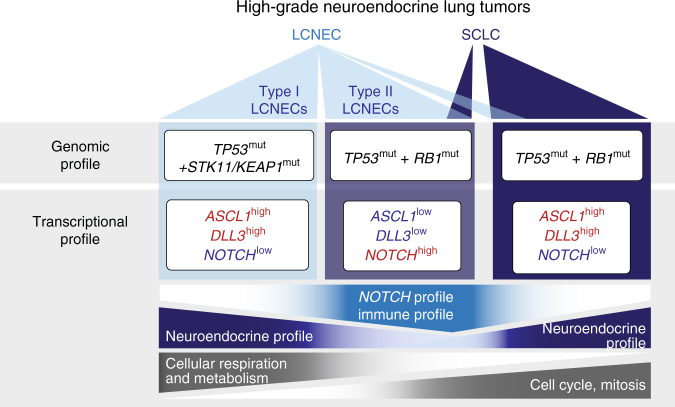
Schematic overview of somatic alterations and expression profiles in high-grade neuroendocrine lung tumors. Significantly mutated genes are shown in black and differentially expressed genes are highlighted in red and blue, describing higher and lower expression, respectively. Upregulated expression profiles and signaling pathways are indicated by color gradients

Conclusively, LCNECs exhibit a distinct expression profile within the spectrum of high-grade neuroendocrine lung tumors, which can further be divided into two subtypes: type I LCNECs with high neuroendocrine expression and, similar to SCLC, a profile of *ASCL1*^high^/*DLL3*^high^/*NOTCH*^low^, and type II LCNECs with reduced expression of neuroendocrine genes and a pattern of *ASCL1*^low^/*DLL3*^low^/*NOTCH*^high^ (Fig. 4).

## Discussion

Here we provide the first comprehensive molecular analysis of LCNECs, which allowed distinguishing between two genomic subgroups with specific transcriptional patterns, defined as “type I LCNECs” and “type II LCNECs” (Fig. 4).

Type I and II LCNECs harbor key genomic alterations and oncogenic mutations, which are commonly found in SCLC, lung adenocarcinoma or squamous cell carcinoma (e.g., in *RAS* genes, *BRAF*, *NFE2L2*, as well as in *STK11* and *KEAP1* in the case of type I LCNECS, and *RB1* losses in the case of type II LCNECs). One possible explanation for this observation might be a high level of intra-tumor heterogeneity, combined with occurrence of two tumor types in a single tumor. However, the key alterations that we found in LCNECs were mostly clonal, with limited genomic intra-tumor heterogeneity. Furthermore, thorough comparisons of gene expression profiles did not suggest similarities between LCNECs and lung adenocarcinomas or squamous cell carcinomas. Thus, the combinations of distinct sets of mutations with specific patterns of gene expression supports the view that LCNECs are not a variant of the other types of lung cancer, but represent a distinct subgroup within the spectrum of neuroendocrine lung tumors.

In a more focused comparison with the most frequent neuroendocrine type of lung cancer, SCLC, type I LCNECs with *STK11* and *KEAP1* alterations exhibited a high degree of similarity with these carcinomas, as well as high expression of neuroendocrine genes and a profile of *ASCL1*^high^/*DLL3*^high^/*NOTCH*^low^. By contrast, type II LCNECs with *RB1* alterations revealed reduced expression of neuroendocrine genes and a pattern of *ASCL1*^low^/*DLL3*^low^/*NOTCH*^high^. Notch family members play a multifaceted role in the development of neuroendocrine tumors with cell-type specific tumor suppressor and oncogenic functions^40^. We have shown in earlier studies that *NOTCH* serves as a tumor suppressor in SCLC^6^, which mostly harbor high-level expression of the negative regulator of Notch, *DLL3*^6,37,41^ (Fig. 4). A recent clinical trial with an antibody-drug conjugate targeting the non-canonical inhibitory NOTCH ligand, Dll3, has shown early signs of clinical activity in SCLC^37,41^. We now demonstrate shared neuroendocrine pathways between SCLC and type I LCNECs, which may be similarly susceptible to this agent. On the other hand, type II LCNECs with alterations in *RB1* exhibited active Notch signaling (Fig. 4). Clinical trials have assessed the efficacy of an antibody targeting Notch 2 and 3 in SCLC, but recently failed in demonstrating a clinical benefit^42,43^. Therefore, future clinical trials involving therapeutics, targeting activating or inhibitory members of the Notch pathway will—in our view—require clear assignment of the respective molecular subtype.

Perhaps another noteworthy finding, type II LCNECs exhibited a pattern of gene expression with upregulation of immune related pathways (Fig. 3b, Fig. 4), which has similarly been observed in various other tumor types^28^ and which may impact the response of patients to immunotherapy. Taken together, the precise distinction of high-grade neuroendocrine tumors representing as type I LCNECs and as *RB1-*mutated SCLC or type II LCNECs, may be pivotal to assess the efficacy of targeted therapeutics, including Notch pathway and immune checkpoint inhibitors.

Our sequencing studies did not reveal any somatic events that may cause the transcriptional discrepancy observed in LCNEC and SCLC tumors with *TP53* and *RB1* alteration, which raises the question if all neuroendocrine tumors share the same cell of origin. It remains to be understood whether distinct tumor-specific cell of origins or cellular processes allow for plasticity and trans-differentiation that consequently lead to distinct molecular phenotypes. Importantly, histological trans-differentiation from lung adenocarcinoma to SCLC has been observed, both spontaneously or as resistance mechanisms to kinase inhibitors^44,45^; in some cases these were linked with a loss of *RB1*^4,46^. Previous studies involving genetically engineered mouse models and human cell lines have emphasized the phenomenon of transcriptional heterogeneity in SCLC and pointed to discordant expression of key lineage factors (e.g. *ASCL1*, *NEUROD1*, *REST*)^31,38^. By contrast, human primary tumors revealed a more complex expression pattern with co-expression of these transcriptional regulators. As a limitation of bulk tumor sequencing, advances in single cell sequencing may further aid to resolve and study the level of transcriptional intra-tumor heterogeneity in high-grade neuroendocrine tumors. While our studies pointed to transcriptional correlates of genomically defined subsets in LCNECs (type I and type II LNCECs), additional analyses on a larger dataset are warranted to further interrogate subcategories of high-grade neuroendocrine tumors.

In summary, we provide the first comprehensive characterization of neuroendocrine lung tumors, which integrates the molecular phenotypes of less frequent lung tumor subtypes. Despite the fact that LCNEC and SCLC tumors share some common clinical and histological characteristics, our study emphasizes pronounced differences in the pattern of genomic alterations and in their transcriptome profiles. The precise distinction of type I and type II LCNECs from SCLC is consequently pivotal to evaluate the response of patients to treatment options and to further understand morphological trans-differentiation processes in lung cancer patients.

## Methods

### Human specimens

The institutional review board (IRB) of the University of Cologne approved this study. Patient samples were obtained under IRB-approved protocols following written informed consent from all human participants. We collected and analyzed fresh-frozen samples of 75 LCNEC patients, which were provided by multiple collaborating institutions; 42 tumors were previously subject of other studies conducted by Rousseaux et al.^47^ (*n* = 25) and Seidel et al.^7^ (*n* = 37) (Supplementary Data 1). Clinical data were available for most patients, who were predominantly male (approximate ratio of 4:1) and current or former heavy smokers (Supplementary Data 1). All tumor samples were reviewed and confirmed by independent expert pathologists (E.B., W.T., and R.B.), and the diagnosis of LCNEC and the assessment of combined histological components were confirmed by H&E staining and immunohistochemistry, including markers for chromogranin A, synaptophysin, CD56 and Ki67. All tumors were positive for at least one neuroendocrine differentiation marker (Supplementary Data 1–2). Specimens containing >70% of tumor cells were processed for DNA and RNA extractions. DNA was extracted from matching normal material that was provided in the form of blood or adjacent non-tumorigenic lung tissue, which through pathological evaluation was confirmed to be free of tumor contaminants.

### Nucleic acid extraction

Total DNA and RNA were obtained from fresh-frozen tumor tissue and matched fresh-frozen normal tissue or blood. Depending on the size of the tissue, 15–30 sections, each 20 μm thick, were cut using a cryostat (Leica) at –20 °C. The matched normal sample obtained from frozen tissue was processed the same way. Nineteen LCNEC cases were identified with mixed histological components of SCLC, lung adenocarcinomas and squamous cell carcinomas (Supplementary Data 1); in these cases nucleic acids were extracted from pure LCNEC regions by only dissecting the LCNEC component. DNA was extracted with the Gentra Puregene DNA extraction kit (Qiagen) and diluted to a working concentration of 100 ng/μL. The DNA was analyzed by agarose gel electrophoresis and confirmed to be of high-molecular weight (>10 kb). The DNA of tumor and normal material was confirmed to originate from the same patient by short tandem repeat (STR) analysis which was conducted at the Institute of Legal Medicine at the University of Cologne (Cologne, Germany), or by subsequent Affymetrix 6.0 SNP array and sequencing analyses.

RNA was isolated from tumor tissues by first lysing and homogenizing tissue sections with the Tissue Lyzer (Qiagen). The RNA was then extracted with the Qiagen RNAeasy Mini Kit. The RNA quality was analyzed at the Bioanalyzer 2100 DNA Chip 7500 (Agilent Technologies) and cases with a RNA integrity number (RIN) of over seven were considered for RNA-seq experiments.

### Next-generation sequencing (NGS)

WES was performed by first fragmenting 1 μg of DNA (Bioruptor, diagenode, Liége, Belgium). The DNA fragments were then end-repaired and adaptor-ligated with sample index barcodes. Following size selection, the SeqCap EZ Human Exome Library version 2.0 kit (Roche NimbleGen, Madison, WI, USA) was used to enrich for the whole exome. The DNA libraries were then sequenced with a paired-end 2 × 100 bp protocol aiming for an average coverage of 90× and 120× for the normal and tumor DNA, respectively. The primary data were filtered for signal purity with the Illumina Realtime Analysis software.

WGS was performed with a read length of 2 × 100 bp. The samples were processed to provide 110 Gb of sequence, thus amounting to a mean coverage of 30× for both tumor and matched normal.

For RNA-seq, cDNA libraries were prepared from PolyA + RNA following the Illumina TruSeq protocol for mRNA (Illumina, San Diego, CA, USA). The libraries were sequenced with a paired-end 2 × 100 bp protocol resulting in 8.5 Gb per sample, and thus in a 30× mean coverage of the annotated transcriptome.

Whole genome, whole exome and transcriptome sequencing reactions were performed on an Illumina HiSeq 2000 sequencing instrument (Illumina, San Diego, CA, USA).

### Copy-number analysis by Affymetrix SNP 6.0 arrays

Human DNA from fresh-frozen tumors was analyzed with Affymetrix Genome-Wide Human SNP 6.0 arrays to determine copy-number alterations. Raw copy number data were computed by dividing tumor-derived signals by the mean signal intensities obtained from a subset of normal samples which were hybridized to the array in the same batch. Circular binary segmentation was applied to obtain segmented raw copy numbers^48^. Significant copy-number alterations were assessed with CGARS^49^ at a threshold of *Q* < 0.01 (Supplementary Data 4).

### Data processing and analyses of DNA sequencing data

The sequencing reads were aligned to the human reference genome NCBI build 37 (NCBI37/hg19) with BWA (version 0.6.1-r104)^50^. Possible PCR-duplicates were masked and not included for subsequent studies. We applied our in-house analysis pipeline^4,6,51^ to analyze the data for somatic mutations, copy number alterations and genomic rearrangements. In brief, the mutation calling algorithm considers local sequencing depth, forward-reverse bias, and global sequencing error, to thus determine the presence of a mutated allele. We determined the somatic status of these mutations by assessing the absence of these variants in the sequencing data of the matched normal.

We determined genomic rearrangements from WGS data of 11 human LCNECs following the procedure as previously described^6,51^. In brief, the sequencing data were analyzed for discordant read-pairs, which were not within the expected mapping distance (>600 base pairs) or which revealed an incorrect orientation. Discordant read-pairs were analyzed for breakpoint-spanning reads, in which one read-pair shows partial alignments to two distinct genomic loci. Rearranged genomic loci were then reported at instances where at least one breakpoint-spanning read was identified. The genomic rearrangements called from each tumor sample were further filtered against the sequencing data of a matched normal and additionally against a library of normal genomes to thus minimize the detection of false-positive rearrangements.

Significantly mutated genes were analyzed as previously described^4,6^. In brief, we first determined the overall background mutation rate of each gene by computing its expected number of mutations assuming that all mutations are uniformly distributed across the genome. We also considered the ratio of synonymous to non-synonymous mutations into a combined statistical model to determine significantly mutated genes. Since mutation rates in non-expressed genes are often higher than the genome-wide background rate, we furthermore filtered for the expression of genes by referring to the transcriptome sequencing data of LCNECs. Only genes with a median FPKM (Fragments Per Kilobase Million) value of >1 in at least 35 out of 60 samples were considered (Methods section: RNA sequencing data processing and analyses). The significance of recurrently mutated genes was determined at a *Q*-value of <0.01 (Supplementary Data 7). Following previously described methods, we furthermore analyzed the data for significant enrichment of damaging mutations (including splice site, non-sense, and frameshift mutations)^6^ and for significant clustering of mutations in genomic hotspots following a re-sampling based approach^4^. Significance was determined at a *Q*-value of 0.01, if the gene was affected in >10% of the samples (Supplementary Data 7). The damaging impact of mutations was further assessed by Polyphen^52^.

The clonal status of mutations was assessed by computing for every mutation the “cancer cell fraction” (CCF), which defines within a tumor the fraction of cancer cells harboring that particular mutation^53^. The CCF was computed following our previously described approach^6^. In brief, this method first estimates tumor purity, ploidy, and absolute copy numbers, and computes for each mutation in a given sample the expected allele frequency under the assumption of clonality. The CCF is the quotient of the observed allelic fraction and the expected allelic fraction of a mutation. The distribution of CCFs for every mutation in a sample allowed to further identify distinct clusters and to thus assign the mutations to clonal and subclonal populations. The analysis described in Supplementary Fig. 2c considers mutations, which were assigned to clonal and subclonal fractions with a probability >90%. In consideration of the sequencing coverage and the overall distribution of CCFs of every mutation in a sample, we furthermore determined the significant enrichment of mutations in a subclone at a *P*-value of 0.01 (Fig. 1b).

### Mutational signatures analyses

Mutational signatures were analyzed in lung cancer subtypes applying previously described methods^54,55^ and referring to the datasets of 77 lung adenocarcinomas (50 heavy-smokers (hs) and 27 non-smokers (ns) from the TCGA project)^2,25^, 52 lung squamous cell carcinomas (from the TCGA project)^3^, 109 SCLC^6^, and 60 LCNECs from this study. Tumor cases with at least 30 somatic variants were selected and the list of variants were either extracted from Supplementary Materials^6^ or COSMIC v68 (for the TCGA data)^20^. Variants were annotated with Annovar (version 12 Nov 2014). Gene strand orientations were retrieved from the RefSeqGene database using a customized Perl script. Variants were included in the analyses only if they could be successfully annotated. Single-base substitutions were classified into 96 types determined by the six possible substitutions (C:G > A:T, C:G > G:C, C:G > T:A, A:T > C:G, A:T > G:C, A:T > T:A) in their tri-nucleotides sequence context (16 combinations for each type of substitution). For extracting mutational signatures, we used the non-negative matrix factorization (NMF) algorithm developed by Lee et al.^56^ and implemented in the Welcome Trust Sanger Institute (WTSI) mutational signatures framework.

### Di-deoxynucleotide sequencing

Somatic alterations of interest were determined and confirmed by two independent sequencing approaches, which included WGS, WES, RNA-seq or di-deoxynucleotide sequencing. Di-deoxynucleotide chain termination sequencing (Sanger sequencing) was performed to validate mutations, genomic rearrangements, and chimeric fusion transcripts. Primer pairs were designed to amplify the target region encompassing the somatic alteration. The PCR reactions were performed either with genomic DNA or cDNA. The amplified products were subjected to Sanger sequencing and the respective electropherograms were analyzed by visual inspection using 4 Peaks or Geneious.

### Analysis of RNA sequencing data

In order to detect chimeric transcripts, RNA-seq data were processed using TRUP^4,27^. In brief, paired-end RNA-seq reads were aligned to the human reference genome (NCBI37/hg19). We used TRUP to identify potential chimeric transcripts. Gene expression levels were determined with Cufflinks v2.0.2 referring only to paired-end reads that uniquely mapped within the expected mapping distance. The expression was quantified as FPKM (Fragments Per Kilobase Million) and the expression values served as a filter for identifying significantly mutated genes (Methods section: Data processing and analyses of DNA sequencing data).

### Gene expression profiling and clustering studies

We analyzed transcriptome sequencing data from a total of *n* = 341 lung cancer samples. *N* = 221 samples referred to the data generated at the University of Cologne, Department of Translational Genomics, which included 41 lung adenocarcinoma^26,27^, 61 pulmonary carcinoids^24^, 53 SCLC^6^, and 66 LCNECs from this present study. *N* = 120 samples were randomly selected from both the TCGA lung squamous cell carcinoma (*n* = 60)^3^ and TCGA lung adenocarcinoma (*n* = 60) cohorts^2,25^ referring to the Genomics Data Commons Legacy Archive. Sequencing data of lung adenocarcinomas from two different platforms aided in controlling for potential batch effects in subsequent studies. The raw sequencing reads of the RNA-seq data were all similarly processed to analyze for gene expression profiles. Sequencing reads which passed the quality control were mapped to the human reference genome (hg19) using MapSplice^57^. Picard Tools v1.64 (http://broadinstitute.github.io/picard/) was used to assess the alignment profile. SAMtools was used to sort and index the mapped reads and to determine transcriptome coordinates. The aligned reads were further filtered for indels, large inserts, and zero mapping quality with UBU v1.0 (https://github.com/mozack/ubu). RSEM^58^, an expectation-maximization algorithm that refers to UCSC gene transcript and definitions, was applied to estimate transcript abundance. In order to allow comparisons between all RNA-Seq samples, raw RSEM read counts were normalized to the overall upper quartile^59^. The expression was quantified for 20,500 genes in 341 tumor samples and the median expression value was determined at RSEM = 209, which served as a reference threshold to classify for low and high expression. The expression determined by RSEM is provided for LCNECs in Supplementary Data 11.

For clustering purposes a set of genes that were both highly expressed and had highly variable expression patterns was identified in all lung cancer subtypes. Quality control procedures performed prior to any clustering analysis did not detect any evidence of batch effects.

After median centering the log_2_(RSEM + 1) values by gene, unsupervised consensus clustering was applied using the ConsensusClusterPlus R package^60,61^ with partitioning around medioids and a Spearman correlation-based distance. Additional hierarchical clustering of the consensus clustering classes was performed, applying average linkage and a Pearson correlation-based distance.

The statistical significance of the differences in gene expression patterns present in the subtype was assessed with the SigClust R package^62^ by referring to the clustering gene sets and by using 1000 permutations and the default covariance estimation method. ClaNC^63^ was used to identify genes whose expression patterns characterize the subtypes. R 3.0.2^61^ was used to perform all statistical analyses and create all figures.

We first conducted consensus clustering of all lung cancer subtypes. The expression data of all lung cancer subtypes (*n* = 341) was analyzed and the 0.75 quantile of all log_2_(mean(RSEM)) values was used to identify highly expressed genes, while the 0.9 quantile of log_2_(variance(RSEM)) was used as a threshold to identify clustering gene sets that have highly variable expression patterns, which yielded a set of 1854 genes (Supplementary Fig. 6a). The samples were clustered with ConsensusClusterPlus following partition around medoids (PAM), and the ConsensusClusterPlus output along with gene expression heatmaps, principal components analysis, and silhouette plots was analyzed. Manual review of ConsensusClusterPlus output suggested a possible clustering solution based on *k* = 6 groups. However, two of the six groups included mainly lung adenocarcinoma samples and the gene expression heatmaps and PCA plots showed that these groups were quite similar. Thus, we chose to collapse these groups, thereby producing a five-class solution. The consensus clusters highly correlated with the histological subtypes as determined by Fisher’s exact test Monte Carlo version (*P* < 0.001, 10,000 permutations): class A (*n* = 66; enriched for pulmonary carcinoids), class B (*n* = 65, enriched for lung squamous cell carcinomas), class C (*n* = 108, enriched for lung adenocarcinomas; data generated by different institutes), class D (*n* = 38, enriched for LCNEC and SCLC cases), and class E (*n* = 64, enriched for SCLC and LCNEC cases) (Supplementary Fig. 6b, Supplementary Data 12). ClaNC led to the identification of 875 classifier genes, which are displayed in the expression heatmaps (Fig. 2, Supplementary Fig. 6–7, Supplementary Data 13).

We then conducted consensus clustering of LCNECs, SCLC, lung adenocarcinomas, and squamous cell carcinomas. The unsupervised clustering approach was repeated for a subset of lung cancer subtypes; here excluding pulmonary carcinoids. The feature selection of highly variable (0.75 quantile) and highly expressed (0.9 quantile) genes across these lung tumor subtypes (*n* = 280) involved a gene set of 1855 genes and the consensus clustering process through hierarchical clustering suggested the presence of three expression clusters (expression subtypes): class A (*n* = 98, enriched for lung adenocarcinomas), class B (*n* = 115, enriched for LCNEC and SCLC), and class C (*n* = 67, enriched for lung squamous cell carcinomas). ClaNC identified 300 classifier genes which are displayed in the respective expression heatmaps (Supplementary Fig. 9).

We performed consensus clustering of LCNEC and SCLC through unsupervised clustering of the expression data of LCNEC and SCLC tumors alone (*n* = 119). Exploratory analyses of the gene expression data suggested the use of the 0.9 quantile of both the log_2_(mean(RSEM)) and log_2_(variance(RSEM)) values as thresholds for highly expressed and highly variably expressed genes. This produced a set of 1416 clustering genes. The Consensus clustering approach included hierarchical clustering and yielded four gene expression subtypes: class I (*n* = 19, only LCNECs), class II (*n* = 49, LCNEC and some SCLC tumors), class III (*n* = 10, SCLC and some LCNECs), and class IV (*n* = 41, mainly SCLC and some LCNECs) (Fig. 3, Supplementary Fig. 10–11, Supplementary Data 12). Hierarchical clustering of these cases revealed two main subgroups: one mainly formed by class I and II (enriched for LCNECs) and one mainly formed by class III and IV (enriched for SCLC) (Supplementary Fig. 11). 300 classifier genes were identified by ClaNC and are displayed in the expression heatmaps (Fig. 3, Supplementary Fig. 11, Supplementary Data 13).

We also performed consensus clustering of LCNECs with lung adenocarcinomas or lung squamous cell carcinomas. A gene set of (a) 1335 and (b) 1338 highly variable (0.85 quantile) and expressed genes (0.925 quantile) was identified in subsets of lung cancer tumors, including (a) LCNECs and lung adenocarcinomas (*n* = 167) and (b) LCNECs and lung squamous cell carcinomas (*n* = 126). The consensus clustering approach through PAM (partitioning around medoids) suggested in both cases two transcriptional subclasses: for approach (a) class A (*n* = 70, mainly LCNECs) and class B (*n* = 97, mainly lung adenocarcinomas); and for approach (b) class A (*n* = 58, mainly LCNECs) and class B (*n* = 68, mainly lung squamous cell carcinomas). ClaNC identified 100 classifier genes in each approach, which were used for the expression heatmaps (Supplementary Fig. 9).

We furthermore performed consensus clustering of LCNECs alone. The transcriptional data on LCNECs was analyzed and hierarchical clustering referred to 475 very highly expressed (0.875 quantile) and very highly variable (0.975 quantile) genes. The consensus clustering approach yielded a *k* = 4 clustering solution: class 1 (*n* = 11), class 2 (*n* = 21), class 3 (*n* = 24), and class 4 (*n* = 10). ClaNC was then applied to the clustering solution, which further identified 540 classifier genes (Supplementary Fig. 13, Supplementary Data 13).

### Differential expression analysis

The SAMR R package^64^ was used to identify genes that were differentially expressed in the expression subtypes using 1000 permutations and a *Q*-value threshold of 0.05 (Supplementary Data 13). We then used the DAVID annotation database^65,66^ to identify pathways that were enriched for differentially expressed genes at *P* < 0.0001 (Supplementary Data 14).

### **Immunohistochemistry**

FFPE tissue sections of 3-μm thickness were stained for hematoxylin and eosin (H&E) and immunohistochemistry (IHC) was conducted for CD56 (*NCAM1*), Synaptophysin (*SYP*), Chromogranin A (*CHGA*, clone DAK-A3), TTF-1 (*NKX2-1*, clone 8G7G3/1), and Rb1 (*RB1*, clone 1F8 (ab81701; Abcam, Cambridge, UK) (Supplementary Data 2, Supplementary Table 1). Hematoxylin and eosin (H&E) were scanned and can be viewed online or with the Pannoramic Viewer software (3D Histech) as specified in Supplementary Data 2 (for further information see “Data Availability”).

Specifically, IHC for Rb1 was performed with the Novolink max polymer detection system (RE7280-CE, Leica Biosystems, Wetzlar, Germany) using EDTA buffer pH 8.0 (K038, Diagnostic BioSystems, Pleasanton, USA) antigen retrieval (4 × 5 min by microwave 700 W). The primary antibody was incubated overnight at 4 °C; the secondary antibody was incubated for 30 min at room temperature. The signal was visualized by diaminobenzidine after incubation for 5 min at room temperature. Sections were counter-stained with hematoxylin for 5 min. The *H*-score method was used for evaluating the immunostaining with Rb1 by multiplying the intensity of the staining (0: no staining, 1: weak, 2: moderate and 3: strong staining) with the percentage of the tumor or stroma stained. The minimum score was 0 and the maximum was 300 (Supplementary Data 2).

### Fluorescence in situ hybridization assay

Genomic rearrangements of *PTK6* on chromosome 20 were assessed through a dual-color break-apart fluorescence in situ hybridization (FISH) assay following previous protocols^67^. In brief, the BAC clone RP11-939M14 labeled centromeres with biotin (red signal) and CTD-3228E10 labeled telomeric sites with digoxigenin (green signal). The samples were analyzed with a 63× oil immersion objective at a fluorescence microscope (Zeiss, Jena, Germany) equipped with appropriate filters, a charge-coupled device camera and the FISH imaging and capturing software Metafer 4 (Metasystems, Altlussheim, Germany). Two independent scientists analyzed the experiment (R.M. and S.P.). Translocations were derived from a split of a signal pair, resulting in a single red and green signal, single red or green signals resulting from signal loss, were referred to as a rearrangement through deletion. In cases where cells were wild type and displayed no rearrangements, a juxtaposed red and green signal (mostly forming a yellow signal) was observed.

*NTRK1* break-apart FISH were performed with the ZytoLight SPEC *NTRK1* Dual Color Break Apart Probe (ZytoVision, Bremerhaven, Germany). According to previous protocols^68^, 4 μm sections of FFPE tissue were treated with the Paraffin pretreatment reagent kit (Vysis, Abbott Molecular), and then stained with the probes following the instructions of the manufacturer. An *NTRK1* rearrangement was diagnosed when >15% of the nuclei showed either a split pattern with 3′ and 5′ signals separated by a distance superior to the diameter of the largest signal, or isolated 3′ (orange) signals.

### Data availability

Sequencing data and Affymetrix 6.0 SNP array data are deposited at the European Genome-phenome Archive, which is hosted by the EBI (EGA, http://www.ebi.ac.uk/ega/), under accession number EGAS00001000708. Histological images of FFPE samples from LCNECs of this study are deposited as H&E images (domain 1: https://teleslide.chu-grenoble.fr/ > acces libre > recherche > recherche/TP/LCNEC-study > code access 1793) or as data files compatible with the Pannoramic Viewer software (3D Histech) (domain 2: https://uni-koeln.sciebo.de/index.php/s/xMjs4dqJpqbOVDn); an overview is provided in Supplementary Data 2.

## Electronic supplementary material

Supplementary Information

Description of Additional Supplementary Files

Supplementary Dataset 1

Supplementary Dataset 2

Supplementary Dataset 3

Supplementary Dataset 4

Supplementary Dataset 5

Supplementary Dataset 6

Supplementary Dataset 7

Supplementary Dataset 8

Supplementary Dataset 9

Supplementary Dataset 10

Supplementary Dataset 11

Supplementary Dataset 12

Supplementary Dataset 13

Supplementary Dataset 14

## References

[1] Imielinski M (2012). Mapping the hallmarks of lung adenocarcinoma with massively parallel sequencing. Cell.

[2] Collisson Ea (2014). Comprehensive molecular profiling of lung adenocarcinoma. Nature.

[3] Hammerman PS (2012). Comprehensive genomic characterization of squamous cell lung cancers. Nature.

[4] Peifer M (2012). Integrative genome analyses identify key somatic driver mutations of small-cell lung cancer. Nat. Genet..

[5] Rudin CM (2012). Comprehensive genomic analysis identifies SOX2 as a frequently amplified gene in small-cell lung cancer. Nat. Genet..

[6] George J (2015). Comprehensive genomic profiles of small cell lung cancer. Nature.

[7] Seidel D (2013). A genomics-based classification of human lung tumors. Sci. Transl. Med..

[8] Bhattacharjee a (2001). Classification of human lung carcinomas by mRNA expression profiling reveals distinct adenocarcinoma subclasses. Proc. Natl Acad. Sci. USA.

[9] Hayes DN (2006). Gene expression profiling reveals reproducible human lung adenocarcinoma subtypes in multiple independent patient cohorts. J. Clin. Oncol..

[10] Wilkerson MD (2010). Lung squamous cell carcinoma mRNA expression subtypes are reproducible, clinically important, and correspond to normal cell types. Clin. Cancer Res..

[11] Chen F (2017). Multiplatform-based molecular subtypes of non-small-cell lung cancer. Oncogene.

[12] Travis WD (2010). Advances in neuroendocrine lung tumors. Ann. Oncol..

[13] Travis, W. D. et al. The 2015 World Health Organization Classification of lung tumors. *J. Thorac. Oncol*. **10**, 1243–1260 (2015). 10.1097/JTO.0000000000000630 26291008

[14] Fasano M (2015). Pulmonary large-cell neuroendocrine carcinoma: from epidemiology to therapy. J. Thorac. Oncol..

[15] Karlsson A, Brunnström H, Lindquist KE, Jirström K (2015). Mutational and gene fusion analyses of primary large cell and large cell neuroendocrine lung cancer Patient material. Oncotarget.

[16] Rekhtman N (2016). Next-generation sequencing of pulmonary large cell neuroendocrine carcinoma reveals small cell carcinoma-like and non-small cell carcinoma-like subsets. Clin. Cancer Res..

[17] Miyoshi T (2017). Genomic profiling of large-cell neuroendocrine carcinoma of the lung. Clin. Cancer Res..

[18] Jones MH (2004). Two prognostically significant subtypes of high-grade lung neuroendocrine tumours independent of small-cell and large-cell neuroendocrine carcinomas identified by gene expression profiles. Lancet.

[19] Alexandrov LB, Stratton MR (2014). Mutational signatures: the patterns of somatic mutations hidden in cancer genomes. Curr. Opin. Genet. Dev..

[20] Forbes SA (2014). COSMIC: exploring the world’s knowledge of somatic mutations in human cancer. Nucleic Acids Res..

[21] Campbell JD (2016). Distinct patterns of somatic genome alterations in lung adenocarcinomas and squamous cell carcinomas. Nat. Genet..

[22] Weiss J (2010). Frequent and focal FGFR1 amplification associates with therapeutically tractable FGFR1 dependency in squamous cell lung cancer. Sci. Transl. Med..

[23] Wistuba II, Gazdar AF, Minna JD (2001). Molecular genetics of small cell lung carcinoma. Semin. Oncol..

[24] Fernandez-Cuesta L (2014). Frequent mutations in chromatin-remodeling genes in pulmonary carcinoids. Nat. Commun..

[25] Imielinski M (2012). Mapping the Hallmarks of lung adenocarcinoma with massively parallel sequencing. Cell.

[26] Fernandez-Cuesta L (2014). CD74-NRG1 fusions in lung adenocarcinoma. Cancer Discov..

[27] Fernandez-Cuesta L (2015). Identification of novel fusion genes in lung cancer using breakpoint assembly of transcriptome sequencing data. Genome Biol..

[28] Rooney MS, Shukla Sa, Wu CJ, Getz G, Hacohen N (2015). Molecular and genetic properties of tumors associated with local immune cytolytic activity. Cell.

[29] Augustyn A (2014). ASCL1 is a lineage oncogene providing therapeutic targets for high-grade neuroendocrine lung cancers. Proc. Natl Acad. Sci. USA.

[30] Westerman BA (2007). Basic helix-loop-helix transcription factor profiling of lung tumors shows aberrant expression of the proneural gene atonal homolog 1 (ATOH1, HATH1, MATH1) in neuroendocrine tumors. Int. J. Biol. Markers.

[31] Borromeo MD (2016). ASCL1 and NEUROD1 reveal heterogeneity in pulmonary neuroendocrine tumors and regulate distinct genetic programs. Cell Rep..

[32] Sutherland KD (2011). Cell of origin of small cell lung cancer: inactivation of Trp53 and Rb1 in distinct cell types of adult mouse lung. Cancer Cell.

[33] Park K (2011). Characterization of the cell of origin for small cell lung cancer. Cell Cycle.

[34] Song H (2012). Functional characterization of pulmonary neuroendocrine cells in lung development, injury, and tumorigenesis. Proc. Natl Acad. Sci. USA.

[35] Sugano M, Nagasaka T, Sasaki E (2013). HNF4 a as a marker for invasive mucinous adenocarcinoma of the lung. Am. J. Surg. Pathol..

[36] Snyder EL (2013). Article Nkx2-1 represses a latent gastric differentiation program in lung adenocarcinoma. Mol. Cell.

[37] Saunders LR (2015). A DLL3-targeted antibody-drug conjugate eradicates high-grade pulmonary neuroendocrine tumor-initiating cells in vivo. Sci. Transl. Med.

[38] Lim JS (2017). Intratumoural heterogeneity generated by Notch signalling promotes small-cell lung cancer. Nature.

[39] Kazarian M, Laird-Offringa Ia (2011). Small-cell lung cancer-associated autoantibodies: potential applications to cancer diagnosis, early detection, and therapy. Mol. Cancer.

[40] Ranganathan P, Weaver KL, Capobianco AJ (2011). Notch signalling in solid tumours: a little bit of everything but not all the time. Nat. Rev. Cancer.

[41] Pietanza, M. C. et al. Safety, activity, and response durability assessment of single agent rovalpituzumab tesirine, a delta-like protein 3 (DLL3)-targeted antibody drug conjugate (ADC), in small cell lung cancer (SCLC). *Eur. J. Cancer*. **51**, S712 (2015).

[42] Yen WC (2015). Targeting notch signaling with a Notch2/Notch3 antagonist (Tarextumab) inhibits tumor growth and decreases tumor-initiating cell frequency. Clin. Cancer Res..

[43] Pietanza, M. C. et al. Final results of phase Ib of tarextumab (TRXT, OMP-59R5, anti-Notch2/3) in combination with etoposide and platinum (EP) in patients (pts) with untreated extensive-stage small-cell lung cancer (ED-SCLC). *J. Clin. Oncol*. 33, 7508 (2015).

[44] Zakowski MF, Ladanyi M, Kris MG (2006). EGFR mutations in small-cell lung cancers. N. Engl. J. Med..

[45] Morinaga R (2007). Sequential occurrence of non-small cell and small cell lung cancer with the same EGFR mutation. Lung Cancer.

[46] Niederst MJ (2015). RB loss in resistant EGFR mutant lung adenocarcinomas that transform to small-cell lung cancer. Nat. Commun..

[47] Rousseaux S (2013). Ectopic activation of germline and placental genes identifies aggressive metastasis-prone lung. Cancers.

[48] Venkatraman ES, Olshen AB (2007). A faster circular binary segmentation algorithm for the analysis of array CGH data. Bioinformatics.

[49] Lu X, Thomas RK, Peifer M (2014). CGARS: cancer genome analysis by rank sums. Bioinformatics.

[50] Li H, Durbin R (2010). Fast and accurate long-read alignment with Burrows-Wheeler transform. Bioinformatics.

[51] Fernandez-Cuesta L (2014). Frequent mutations in chromatin-remodelling genes in pulmonary carcinoids. Nat. Commun..

[52] Adzhubei IA (2010). A method and server for predicting damaging missense mutations a. Nature.

[53] McGranahan N (2015). Clonal status of actionable driver events and the timing of mutational processes in cancer evolution. Sci. Transl. Med..

[54] Alexandrov LB (2013). Signatures of mutational processes in human cancer. Nature.

[55] Alexandrov LB (2015). Clock-like mutational processes in human somatic cells. Nat. Genet..

[56] Lee SY, Song HA, Amari SI (2012). A new discriminant NMF algorithm and its application to the extraction of subtle emotional differences in speech. Cogn. Neurodyn..

[57] Wang K (2010). MapSplice: accurate mapping of RNA-seq reads for splice junction discovery. Nucleic Acids Res..

[58] Li B, Dewey CN (2011). RSEM: accurate transcript quantification from RNA-Seq data with or without a reference genome. BMC Bioinforma..

[59] Bullard JH, Purdom E, Hansen KD, Dudoit S (2010). Evaluation of statistical methods for normalization and differential expression in mRNA-Seq experiments. BMC Bioinforma..

[60] Wilkerson MD, Hayes DN (2010). ConsensusClusterPlus: a class discovery tool with confidence assessments and item tracking. Bioinformatics.

[61] R Core Team, R. F. for S. C. *R: A language and environment for statistical computing*. (2014). Available at http://www.r-project.org/

[62] Liu Y, Hayes DN, Nobel A, Marron JS (2008). Statistical significance of clustering for high-dimension, low–sample size dataset. J. Am. Stat. Assoc..

[63] Dabney AR (2005). Classification of microarrays to nearest centroids. Bioinformatics.

[64] Tusher VG, Tibshirani R, Chu G (2001). Significance analysis of microarrays applied to the ionizing radiation response. Proc. Natl Acad. Sci. USA.

[65] Huang DW, Lempicki RA, Sherman BT (2009). Systematic and integrative analysis of large gene lists using DAVID bioinformatics resources. Nat. Protoc..

[66] Huang DW, Sherman BT, Lempicki RA (2009). Bioinformatics enrichment tools: Paths toward the comprehensive functional analysis of large gene lists. Nucleic Acids Res..

[67] Menon R (2013). Somatic copy number alterations by whole-exome sequencing implicates YWHAZ and PTK2 in castration-resistant prostate cancer. J. Pathol..

[68] McLeer-Florin A (2012). Dual IHC and FISH testing for ALK gene rearrangement in lung adenocarcinomas in a routine practice. J. Thorac. Oncol..

