# Supplementary material for: Integrative genomic profiling of large-cell neuroendocrine carcinomas reveals distinct subtypes of high-grade neuroendocrine lung tumors
Source: Nat Commun. 2018 Mar 13;9:1048. doi: 10.1038/s41467-018-03099-x (PMC5849599; doi:10.1038/s41467-018-03099-x)
Supplement: Supplementary file 1 — Supplementary Information [file 41467_2018_3099_MOESM1_ESM.pdf]

Supplementary Figure 1

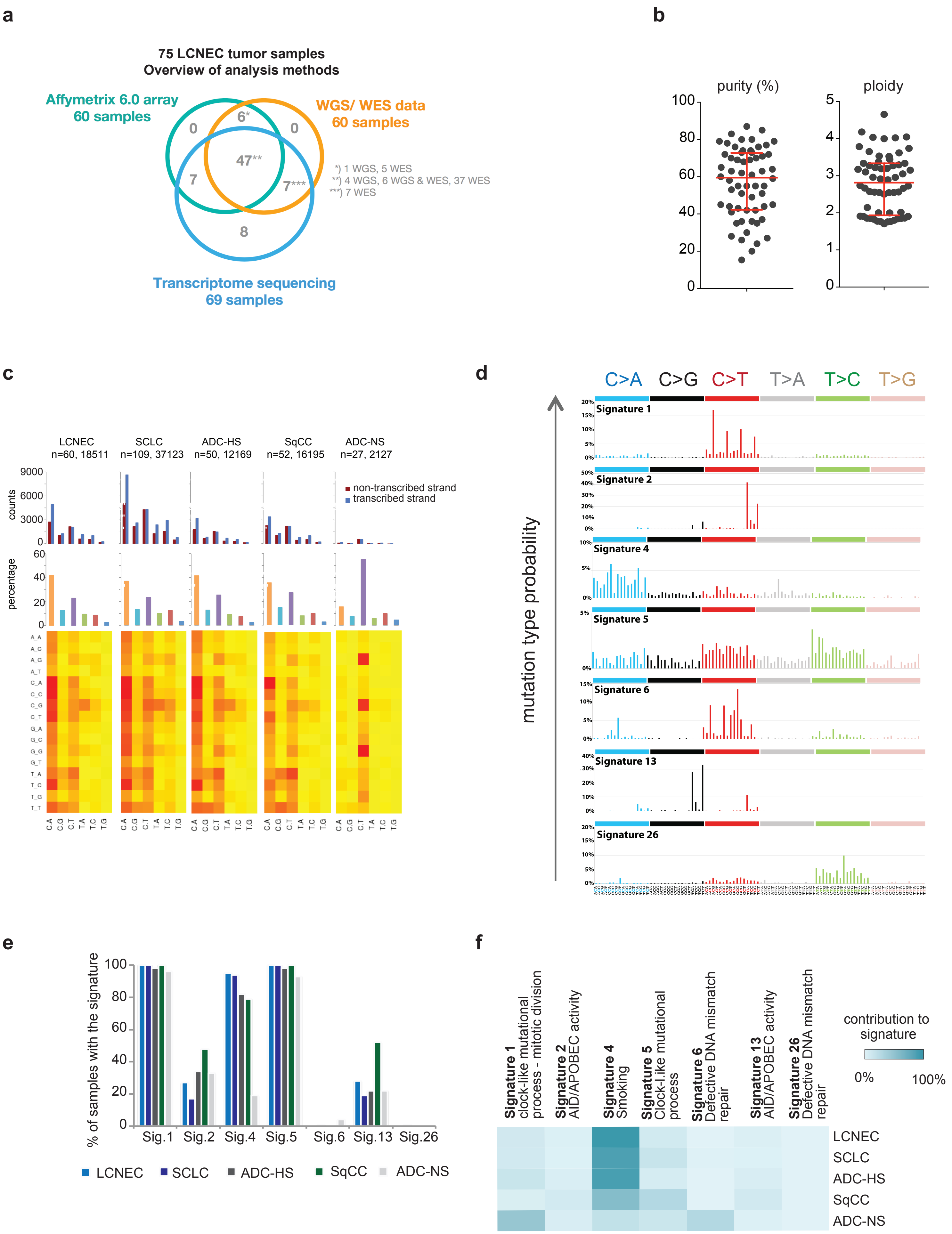

Supplementary Fig. 1. Genomic studies in pulmonary large-cell neuroendocrine carcinomas (LCNECs). (a) Schematic overview of the genomic studies conducted with 75 fresh-frozen LCNEC cases. The number of samples included for each analysis is indicated. (b) Purity and ploidy determined in LCNECs by whole genome and exome sequencing; plots show the median and interquartile range. (c) Distributions of single point mutations on the transcribed and non-transcribed strands (top panels), overall (middle panels), and within the context of their trinucleotide sequence (lower panels) in different lung cancer subtypes. The heatmap is reflecting the percentages. HS: heavy smoker, NS: never smoker. (d) Seven validated mutational signatures identified by negative matrix factorization (NMF), which match those reported for ADC and SqCC[53] and which were recently updated in COSMIC[20]. (e) Percentage of samples of a given lung cancer type harboring each of the seven signatures. (f) Contribution of each of the seven signatures in different lung cancer types.

Supplementary Figure 2

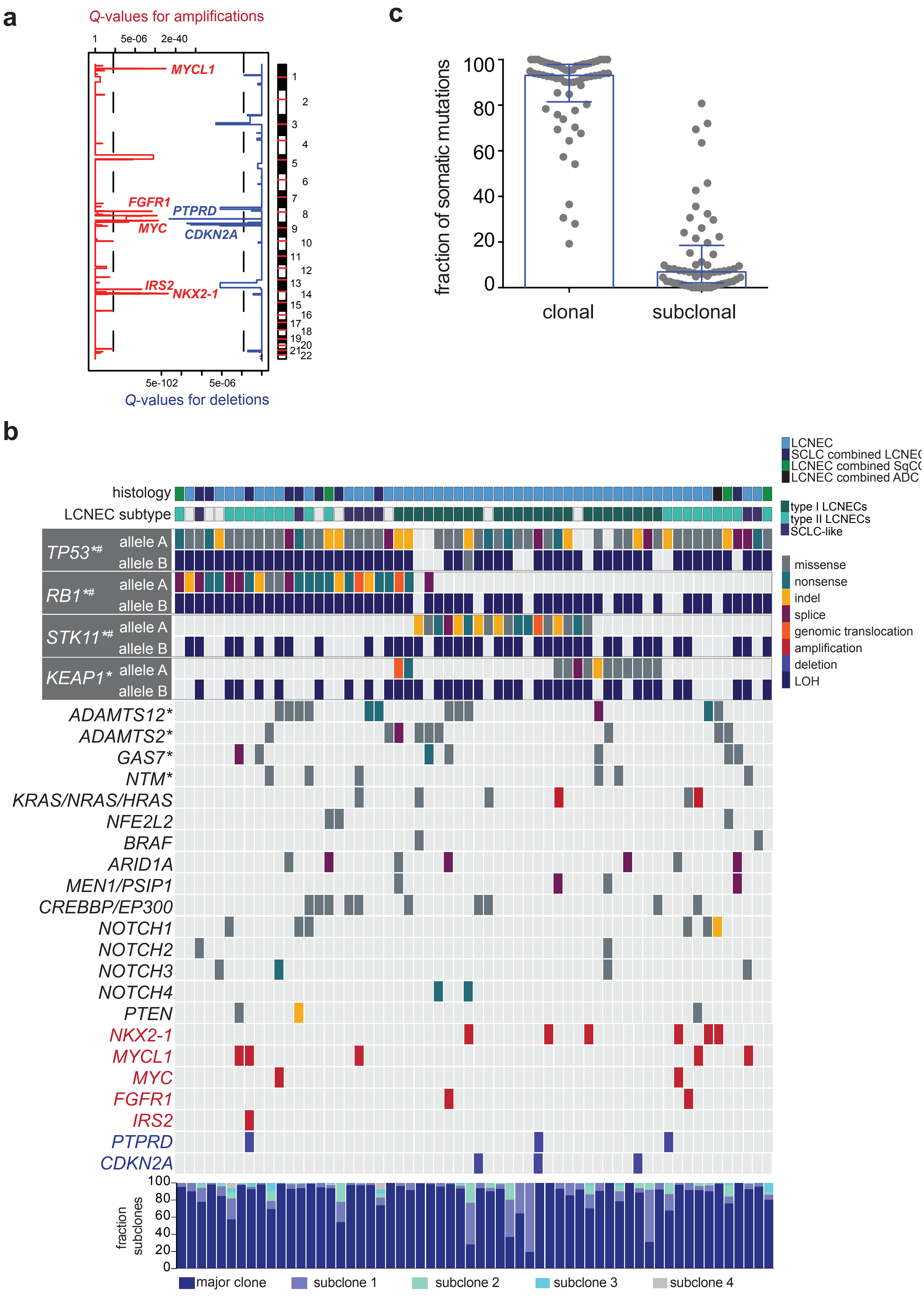

Supplementary Fig. 2. Genomic alterations in pulmonary large-cell neuroendocrine carcinomas (LCNECs). (a) Copy-number analysis to detect significantly altered regions across 60 LCNECs by Affymetrix 6.0 array. Statistical significance is computed for each genomic location and the dashed vertical lines indicate the significance threshold of $Q<0.01$ (Methods section). Deletions (in blue) and amplifications (in red) are analyzed independently. (b) Tumor samples are arranged from left to right. The histological annotation and the assignment to the LCNEC subtype (as defined in the expression clustering approach in Figure 3) are provided according to the color panel. Genomic alterations of candidate genes are annotated for each sample according to the color panel; alterations in *TP53*, *RB1*, *STK11* and *KEAP1* were further specified for alleles A and B, in which larger chromosomal deletions are annotated in dark blue for LOH (loss of heterozygosity). The fraction of clonal and subclonal mutations is displayed in the bottom panel. Significantly mutated genes and significant damaging mutations are denoted with * and #, respectively ($Q<0.01$, Methods section). Genes with significant copy number (CN) amplifications (CN>4) and deletions (CN<1) (Supplementary Fig. 2a, Supplementary Data 5) are displayed according to the color panel in red and blue, respectively ($Q<0.01$, Methods section). (c) The fraction of clonal and sub-clonal mutations was determined for each sample and plotted as bar scatter plots with median and interquartile range.

Supplementary Figure 3

a

		***RB1* alteration**	
		N/A	yes
**Rb IHC score**	0	4	12
	>0	12	0

P < 0.0001

b

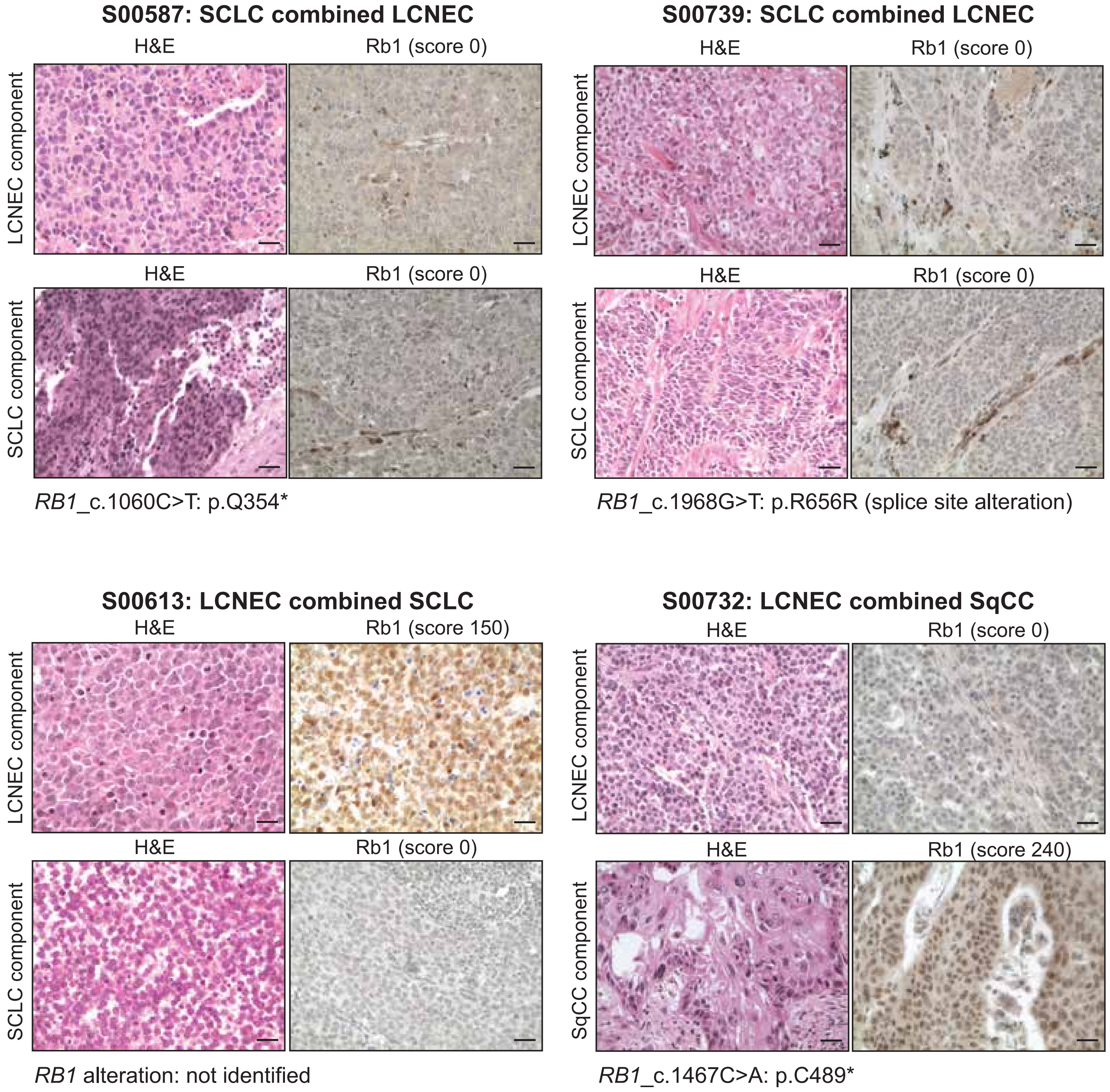

Original magnification, 400x
Scale bar: 25μm

Supplementary Fig. 3. Immunohistochemistry of Rb1 in large-cell neuroendocrine carcinomas. (a) Contingency table for *RB1* alterations and the Rb1 immunohistochemistry (IHC) score determined for 42 LCNECs (Supplementary Data 2). Statistical significance was calculated by Fisher's exact test (P<0.0001). (b) Representative images of hematoxylin and eosin (H&E) and Rb1 IHC stainings of LCNEC cases with admixtures of other histological components (Supplementary Data 2). The genomic status of *RB1* and the IHC score for Rb1 expression is provided for the cases. Original magnification: 400x, Scale bar: 25μm.

Supplementary Figure 4

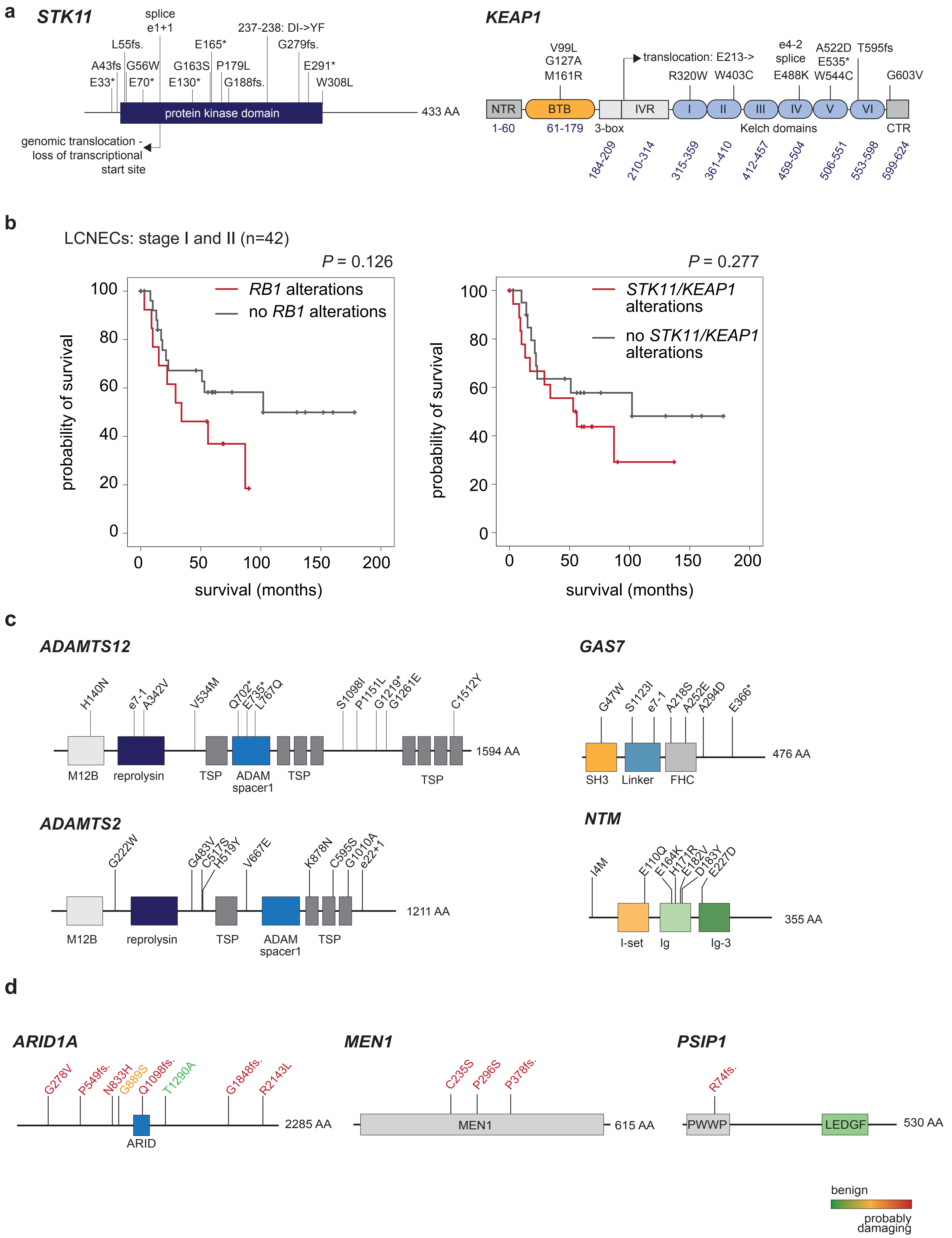

Supplementary Fig. 4. Genomic alterations in key candidate genes and clinical-molecular correlations. (a) Schematic representation of proteins domains affected by genomic alterations in *STK11* and *KEAP1*. NTR: N-terminal region, BTB: Broad complex, Tramtrack and Bric-à-Brac, IVR: intervening region, CTR: C-terminal region. (b) Survival analysis on LCNEC patients (stage I and II) based on the status of *RB1* (left panel) or *STK11* and *KEAP1* alterations (right panel). Statistical significance was determined by log-rank test (P<0.05). (c-d) Schematic representation of proteins for (c) genes with statistically significant enrichment of mutations, and (d) *ARID1A*, *MEN1* and *PSIP1* (Q<0.01, Methods section). Mutations are highlighted according to the color panel indicating the functionally damaging impact as predicted with Polyphen.

Supplementary Figure 5

a

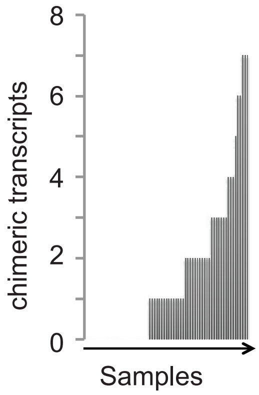

chimeric transcripts	coverage	domains
SMC1A-GPC3	30	Glypican
PIK3R3-CDH12	8	Cadherin
KIAA1432-MMP16	36	Matrixin and hemopexin-x4
ESYT3-FREM1	11	Calx-beta and lectin_C
MSI2-ITGA3	33	RRM_1
ZNF142-PTK6	14	Zinc-finger and tyrosine kinase
RFWD2-NTRK1	22	Zinc-finger and tyrosine kinase

b

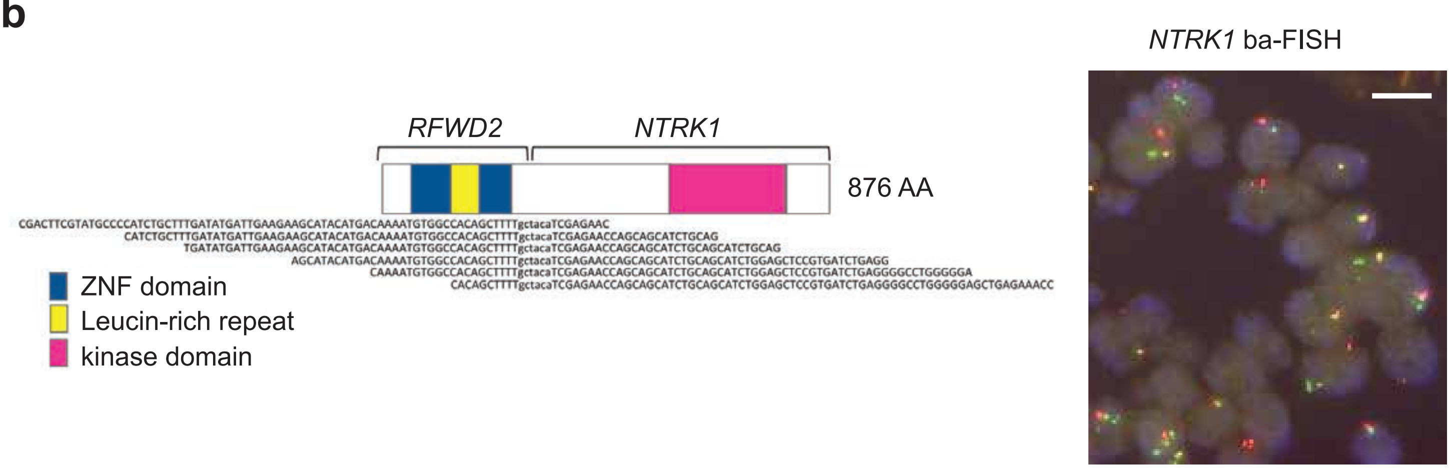

c

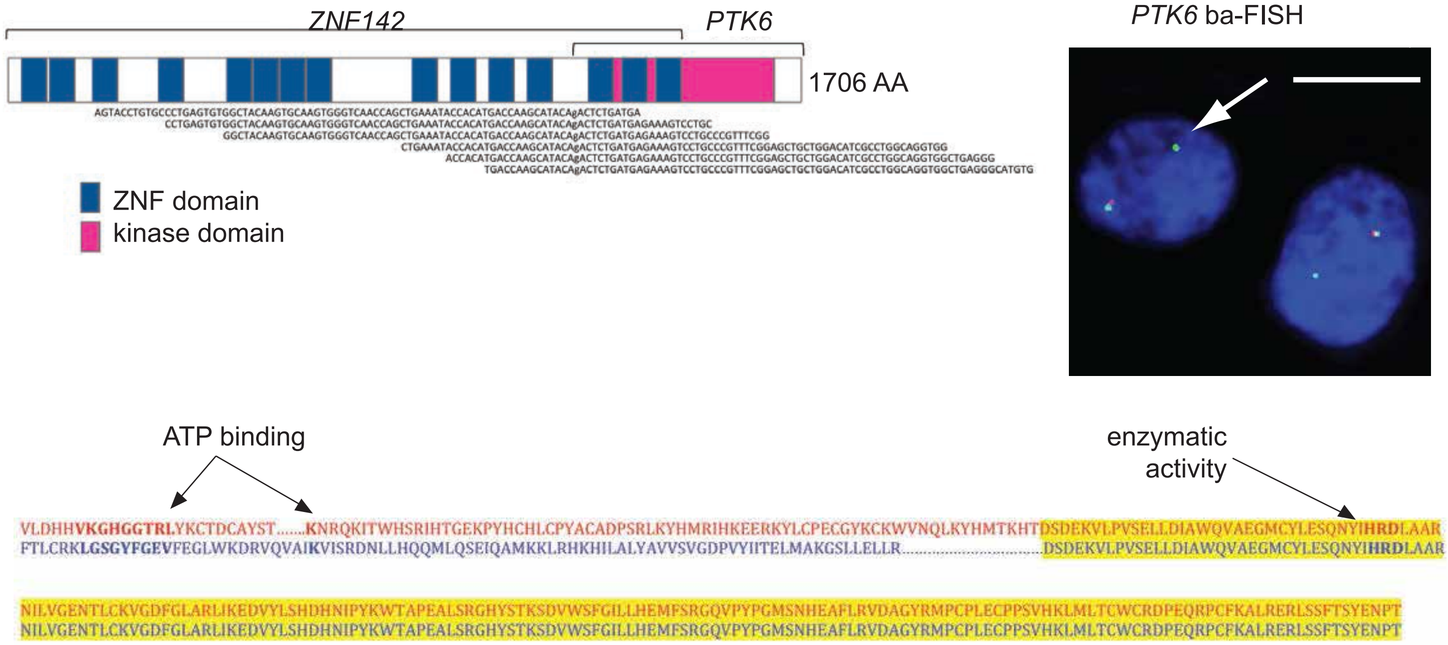

Supplementary Fig. 5: Chimeric fusion transcripts in LCNECs. (a) The left panel shows an overview on the number of chimeric transcripts (y-axis) detected per sample (x-axis). The right panel summarizes a subset of in-frame chimeric transcripts detected in the transcriptome sequencing data of 69 LCNEC samples (Supplementary Data 10). The coverage and protein domains involved are indicated. (b-c) Detection of the (b) *RFWD2-NTRK1* and (c) *ZNF142-PTK6* fusion transcripts by transcriptome sequencing. Schematic representation of the protein domains and some of the sequencing reads spanning the fusion point (left panels). Representative image of the *NTRK1* and *PTK6* break-apart FISH assays are shown in the right panels. (c) The lower panel shows a comparison of the kinase domain sequences of the *ZNF142-PTK6* fusion protein (in red) and the wild-type *PTK6* (in blue), emphasizing the areas coding for the ATP binding and the enzymatic activity of the kinase domains. Highlighted in yellow is the sequence that is conserved in both proteins. Scale bar (white line): 10μm.

Supplementary Figure 6

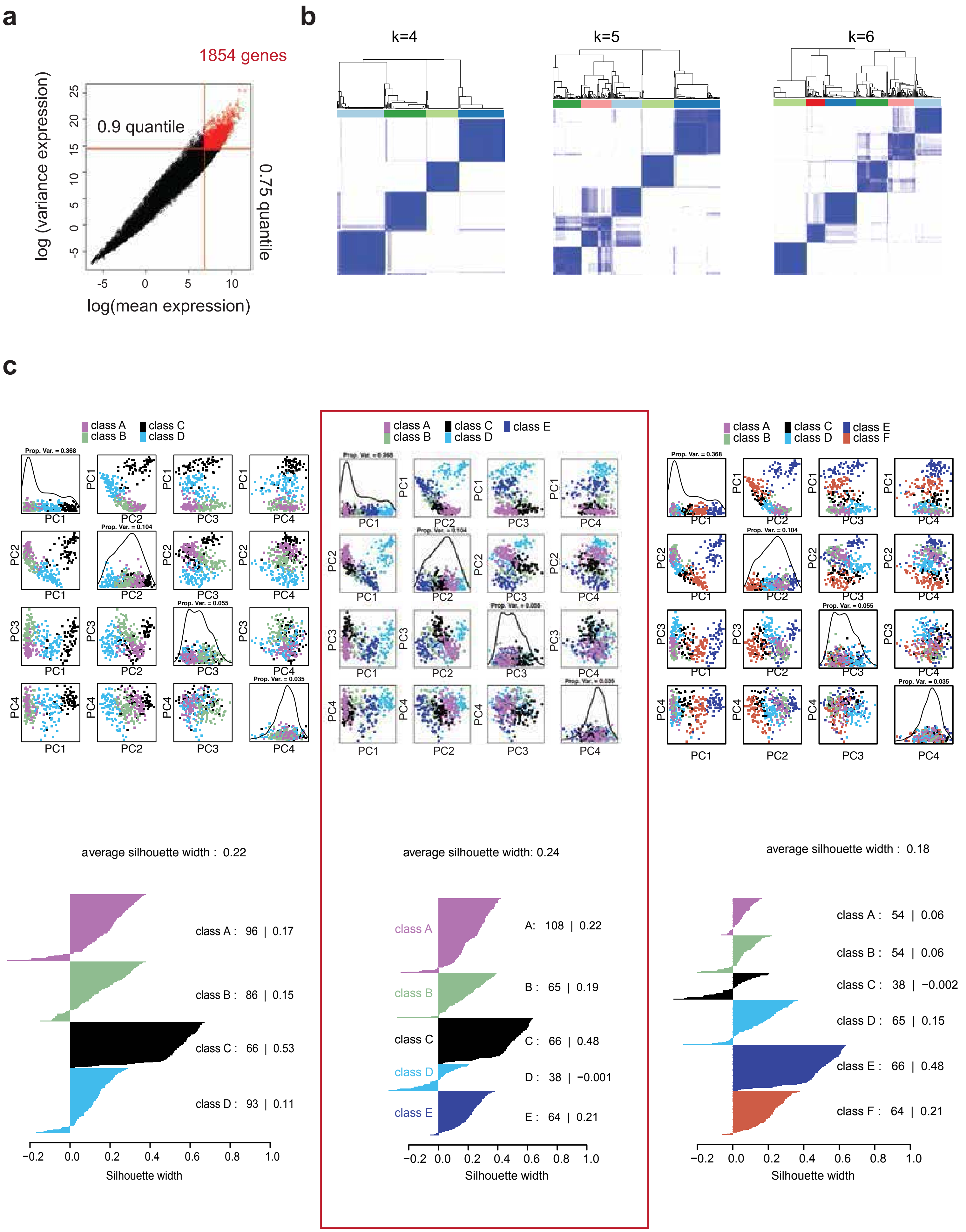

Supplementary Fig. 6. Expression clustering approaches for all lung cancer subtypes. (a) Feature selection for the unsupervised clustering approach was conducted for the transcriptome sequencing data of 101 lung adenocarcinoma, 60 lung squamous cell carcinoma, 61 pulmonary carcinoids, 66 LCNECs and 53 SCLC. 1854 genes with highly variable gene expression across all lung cancer subtypes were selected. (b-c) Consensus cluster plus was applied to all lung cancer subtypes (n=341). The consensus matrix for the cluster solution of k=4, k=5 and k=6 is displayed in (b). Principal component analysis (PCA) and silhouette plots for cluster solutions k=4, k=5 and k=6 are illustrated in (c). Red boxes indicate the clustering solution that was further used in this study.

Supplementary Figure 7

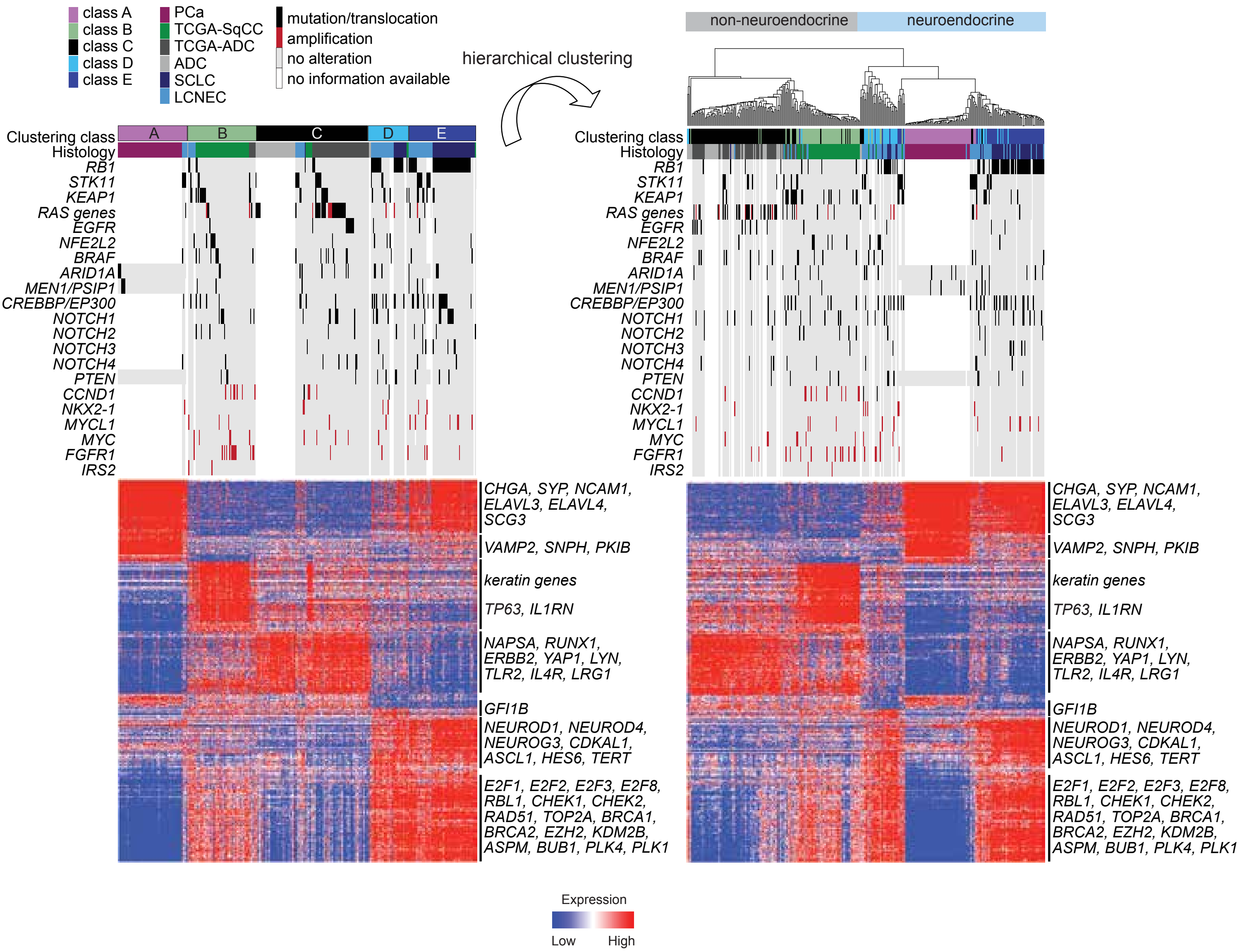

Supplementary Fig. 7. Expression clustering results for all lung cancer subtypes.

The clustering results are displayed as a heatmap referring to ClaNC rank genes (Methods section). Tumor samples are arranged in columns, annotated for the histological subtype and for the somatic alteration status. Selected candidate genes are shown. The samples are grouped according to their expression clustering class (left panel) or according to additional hierarchical clustering of the samples (right panel).

Supplementary Figure 8

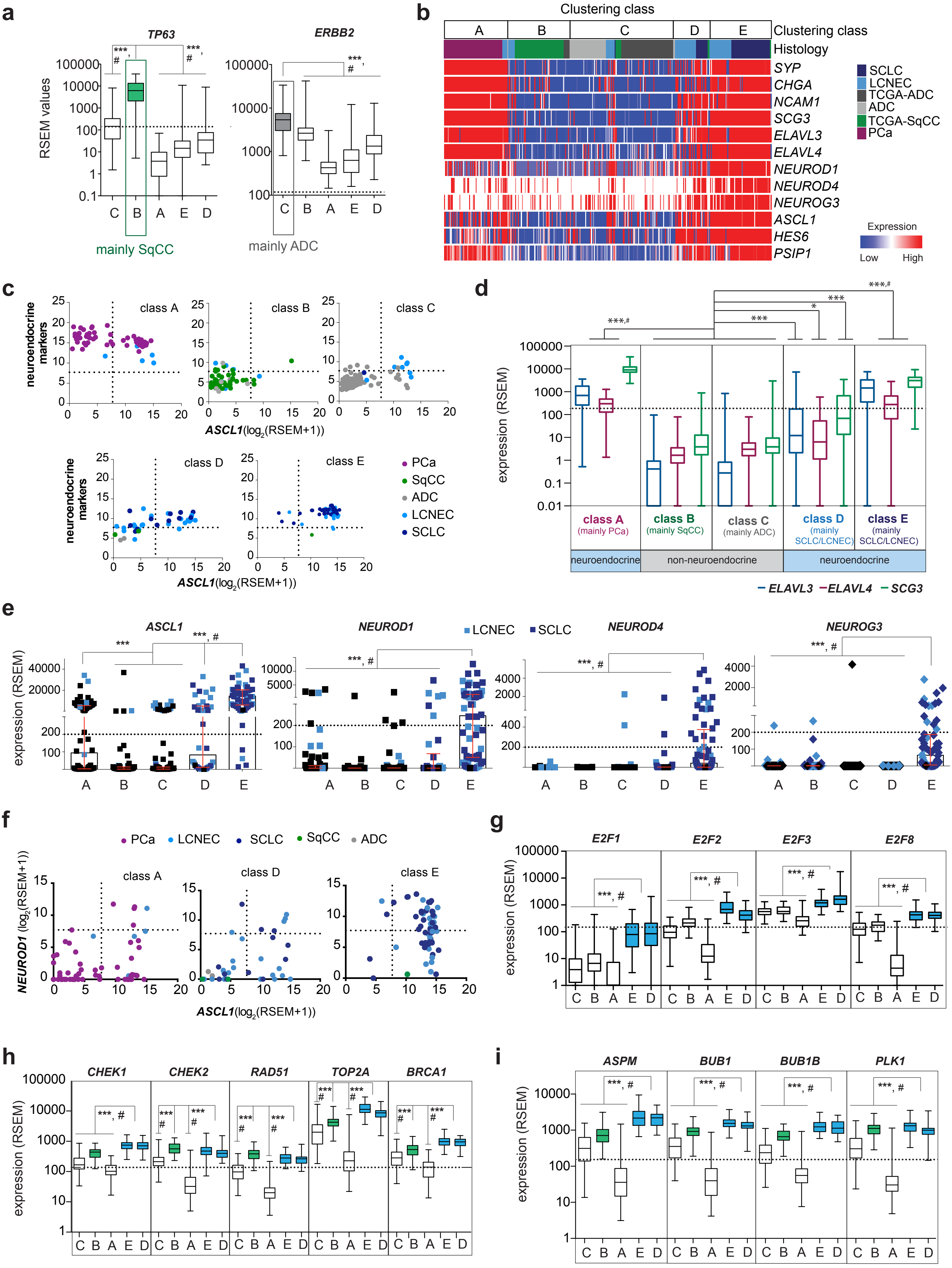

Supplementary Fig. 8: Analysis of differentially expressed genes in lung cancer subtypes. (a) Expression levels of *TP63* and *ERBB2* are displayed as box plots for the clustering classes A-E (median and interquartile range, whiskers: min and max values). Transcriptional class B (mainly lung squamous cell carcinomas) and C (mainly lung adenocarcinomas) are highlighted in green and grey, respectively. (b) Consensus clustering heatmap of selected differentially expressed genes derived from Figure 2a and Supplementary Fig. 7; the histological annotation of the samples is provided according to the color panel. (c) The expression of *ASCL1* and neuroendocrine markers (average value of *SYP*, *NCAM1* and *CHGA*) is illustrated as a scatter plot for each consensus clustering class. The samples are colored according to the histological annotation. (d) Expression levels of *ELAVL3*, *ELAVL4* and *SCG3* are plotted as box plots (median and interquartile range, whiskers: min and max values) for the clustering classes A-E. (e) Expression levels of *ASCL1*, *NEUROD1*, *NEUROD4* and *NEUROG3* for the clustering classes A-E are plotted as scatter bar plot with median and interquartile range. LCNEC and SCLC samples are highlighted according to the color panel. (f) Expression levels of *ASCL1* and *NEUROD1* are illustrated as scatter plots for clustering classes A, E and D, which mainly consist of neuroendocrine lung tumors. The samples are colored according to their histological annotation. (g-i) Expression levels of (g) cell cycle genes, (h) DNA damage genes, and (i) centrosomal genes are displayed for the transcriptional classes A-E as box plots (median and interquartile range, whiskers: min and max values). Classes D and E (enriched for LCNEC and SCLC samples) are highlighted in blue; class C (enriched for lung squamous cell carcinoma samples) is highlighted in green. $Q<0.05$ (#) determined by SAM (Methods section, Supplementary Data 12); $P<0.001$ (***) and $P<0.05$ (*) determined by Mann-Whitney-U test. Dashed black lines indicate the threshold for relative low expression levels (Methods section).

Supplementary Figure 9

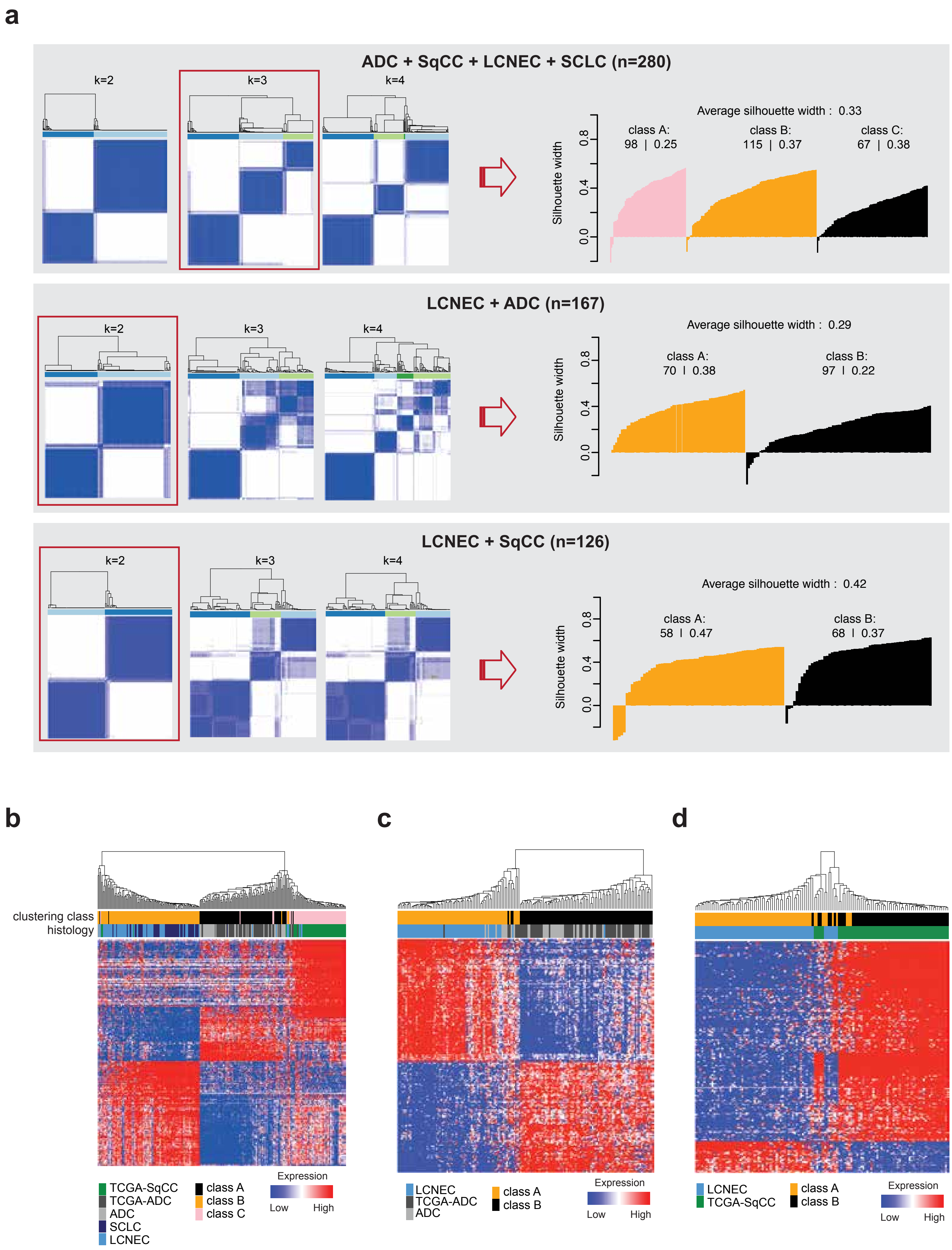

Supplementary Fig. 9. Expression clustering approaches for LCNECs and selected lung cancer subtypes. (a) Transcriptome sequencing data of LCNECs (n=66) were analyzed in combination with lung adenocarcinoma (n=101), lung squamous cell carcinoma (n=60), and SCLC (n=53) (upper panel), or alone with lung adenocarcinomas (middle panel) or lung squamous cell carcinomas (lower panel). The matrix for the consensus cluster solutions of k=2, k=3 and k=4, are displayed on the left, and the cluster solution for subsequent studies is highlighted by red boxes. The right panel displays the silhouette plots of the respective cluster solution. (b-d) The hierarchical clustering solutions are shown as heatmaps referring to ClaNC rank genes.

Supplementary Figure 10

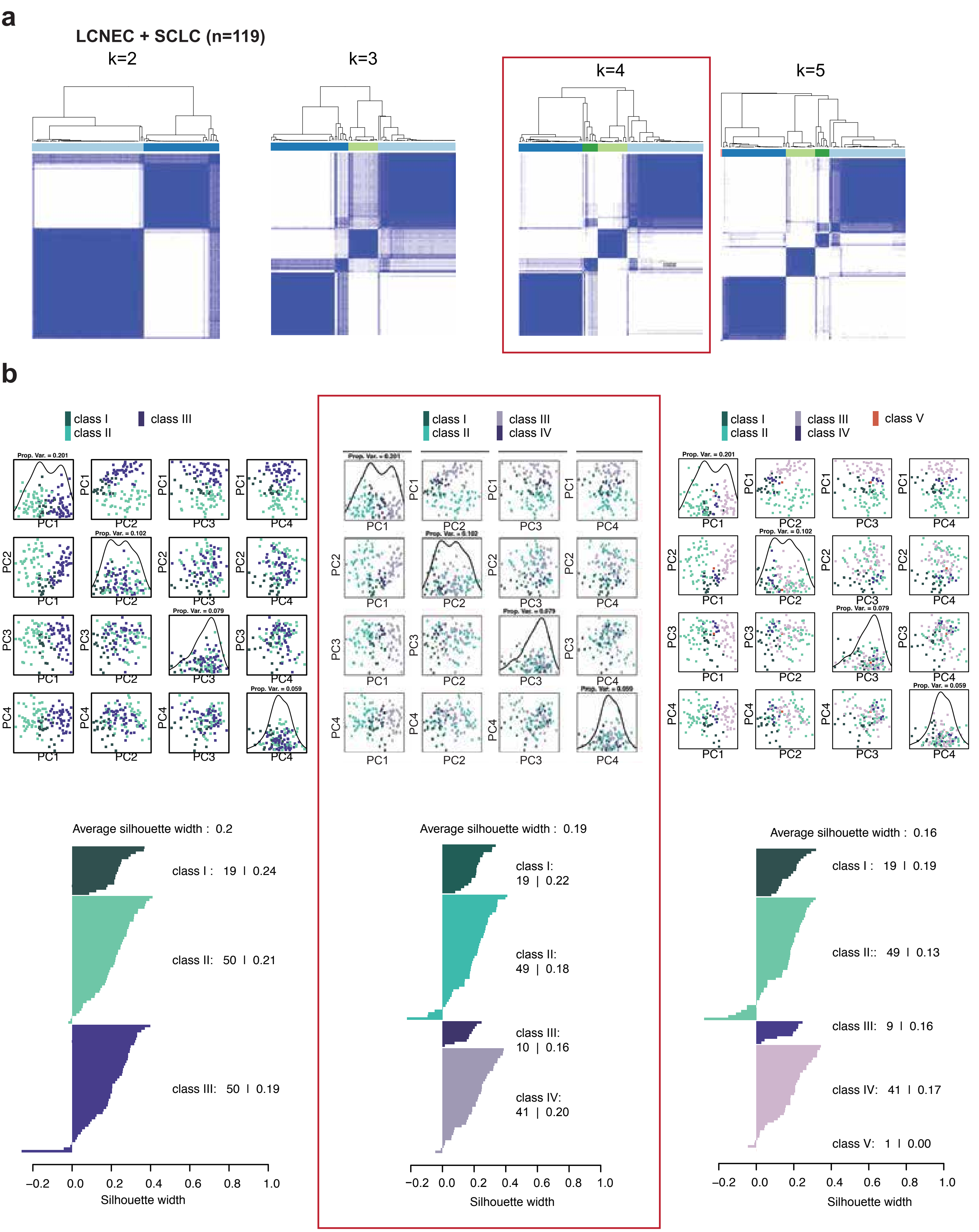

Supplementary Fig. 10: Expression clustering approaches for LCNEC and SCLC. (a) Consensus clustering was applied to the transcriptome sequencing data of 66 LCNECs and 53 SCLC tumors. The matrix for the cluster solutions k=2, k=3, k=4 and k=5 is provided. (b) PCA and silhouette plots for all cluster solutions. Solution k=4 was chosen for subsequent studies (red box).

Supplementary Figure 11

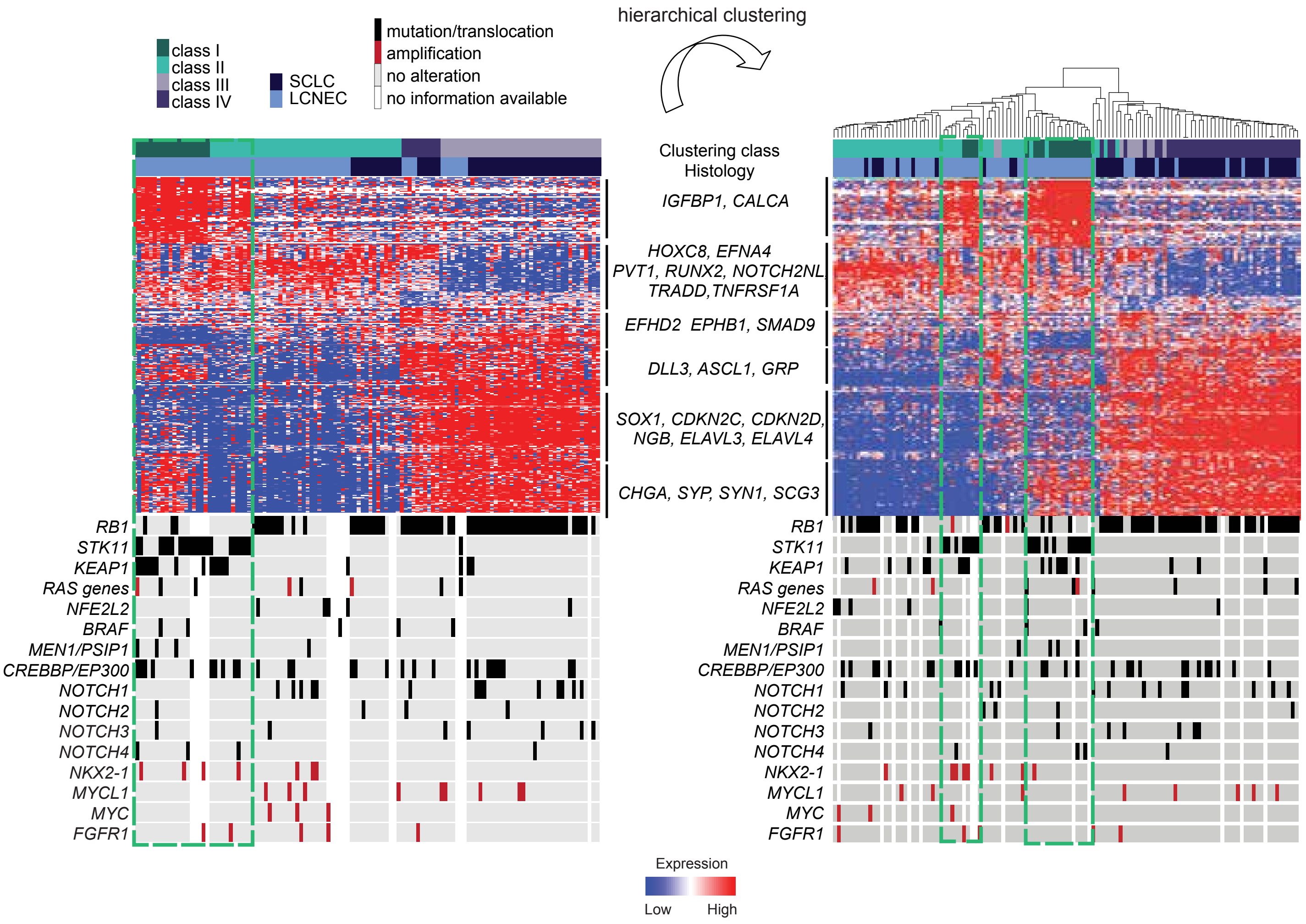

Supplementary Fig. 11: Expression clustering results for LCNEC and SCLC.

The clustering results for SCLC and LCNECs are displayed as a heatmap referring to ClaNC rank genes (Methods section). Tumor samples are arranged in columns, annotated for the histological subtype, for the transcriptional subclasses I-IV, and for the somatic alteration status following the color panel. Selected candidate genes are shown. The samples are grouped according to their expression clustering class (left panel) or according to the hierarchical clustering result (right panel). Genomic alterations identified in these tumors are annotated below the expression heatmap. Dashed green lines indicate an expression profile shared by LCNECs with *STK11*/*KEAP1* alterations (type I LCNECs).

Supplementary Figure 12

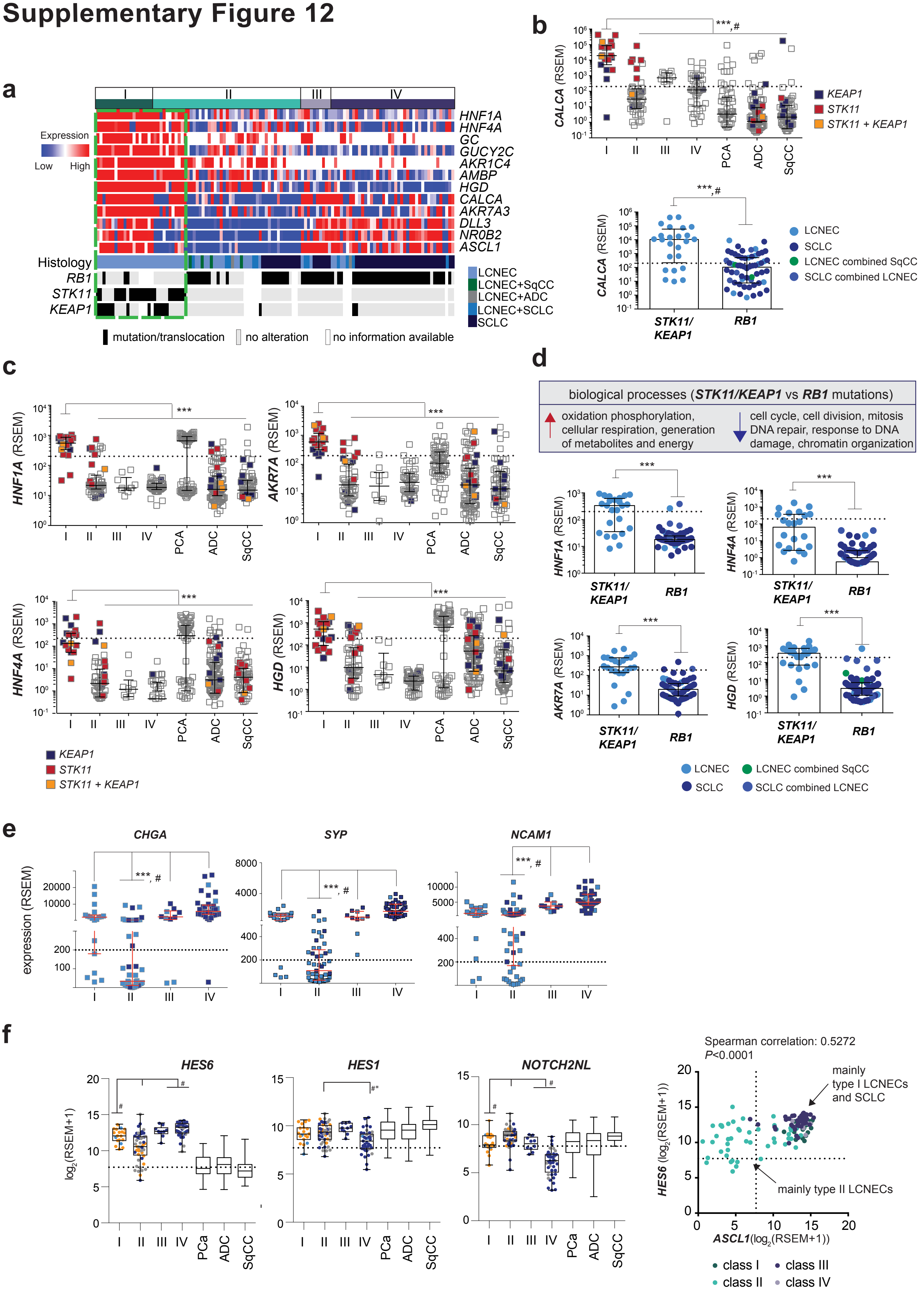

Supplementary Fig. 12: Analysis of differentially expressed genes in transcriptional subsets formed by LCNEC and SCLC. (a) Consensus clustering heatmap of selected differentially expressed genes derived from Figure 3a and Supplementary Fig. 11. The annotation for histology and the somatic alterations status is provided below the heatmap. Dashed green box indicates tumor samples with *STK11* and *KEAP1* alterations. (b) Expression analysis of *CALCA*. The upper panel displays the expression of *CALCA* for the transcriptional classes I-IV (formed by LCNEC and SCLC tumors) in comparison to pulmonary carcinoids, lung adenocarcinomas and squamous cell carcinomas (error bars: median and interquartile range). The bottom panel shows the expression levels in LCNEC and SCLC tumors with *STK11*/*KEAP1* and *RB1* alterations (error bars: median and interquartile range). (c) Expression of metabolism related genes and lineage transcription factors (*HNF1A*, *HNF4*, *AKR7A* and *HGD*) for the transcriptional classes I-IV in comparison to pulmonary carcinoids, lung adenocarcinomas and lung squamous cell carcinomas (error bars: median and interquartile range). Samples with *STK11* and *KEAP1* alterations are highlighted according to the color code. (d) Supervised analysis of differentially expressed genes and pathways in SCLC and LCNECs with *STK11*/*KEAP1* alterations versus tumors with *RB1* alterations. Differentially enriched biological pathways are listed in the top panel. Differential expression of *HNF1A*, *HNF4A*, *AKR7A* and *HGD* is plotted below (error bars: median and interquartile range). The histology of the tumors is highlighted following the color code. (e) Expression of the neuroendocrine markers *CHGA*, *SYP* and *NCAM1* in transcriptional classes I-IV (error bars: median with interquartile range). (f) Expression of the *NOTCH* pathway genes *NOTCH2NL*, *HES6* and *HES1* displayed for the transcriptional classes I-IV in comparison to pulmonary carcinoids, lung adenocarcinomas and squamous cell carcinomas (boxplots: median and interquartile range; whiskers: min and max values). Samples are colored according to their somatic alteration status of *STK11*, *KEAP1* or *RB1*.

Right panel: *ASCL1* and *HES6* expression for each sample is illustrated as a scatter plot (right panel). *Q*<0.05 (#) determined by SAM (Methods section, Supplementary Data 12); *P*<0.001 (***) and *P*<0.05 (*) determined with Mann-Whitney-U test. Dashed black lines indicate the threshold for relatively low expression (Methods section).

Supplementary Figure 13

a

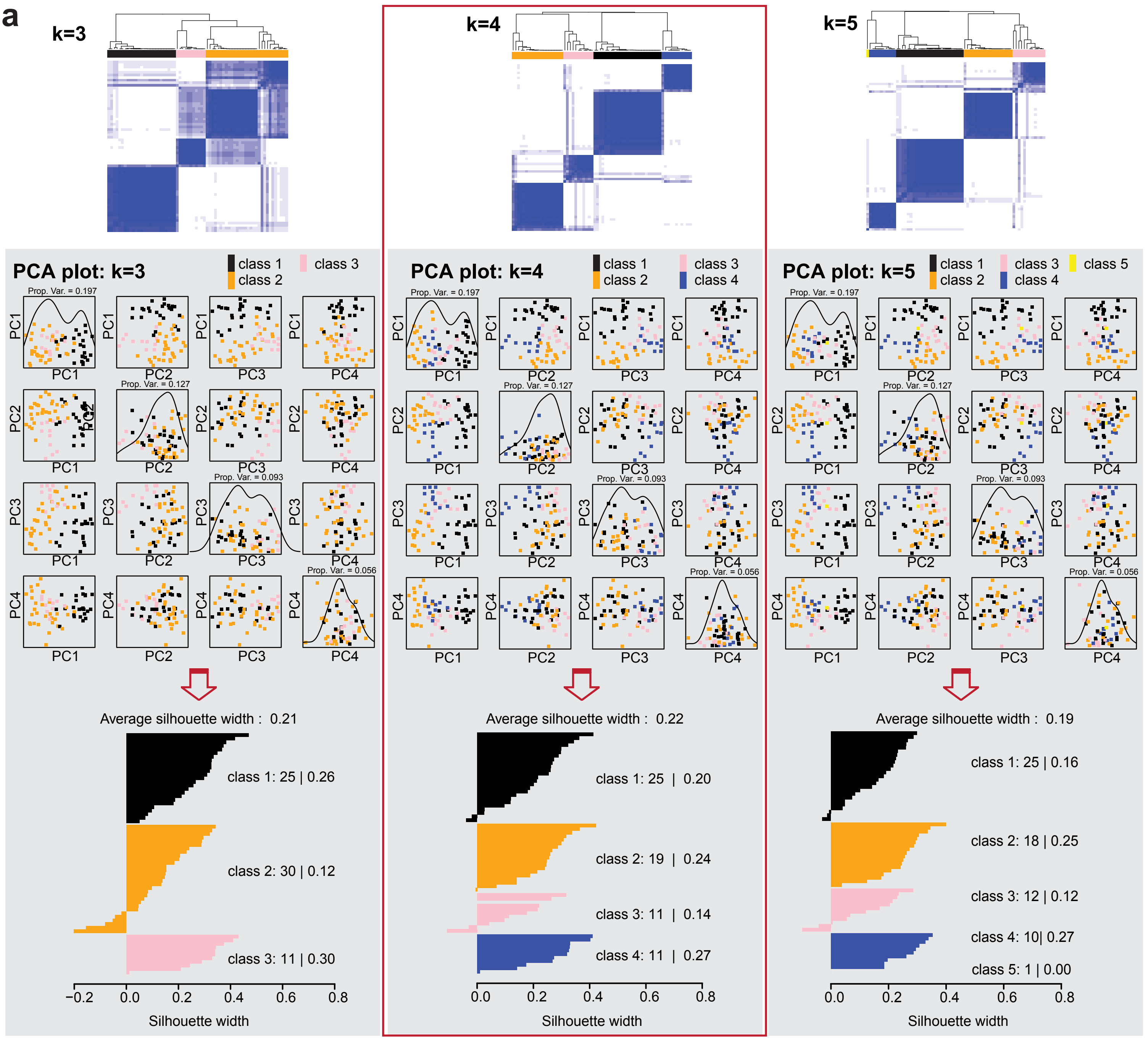

b

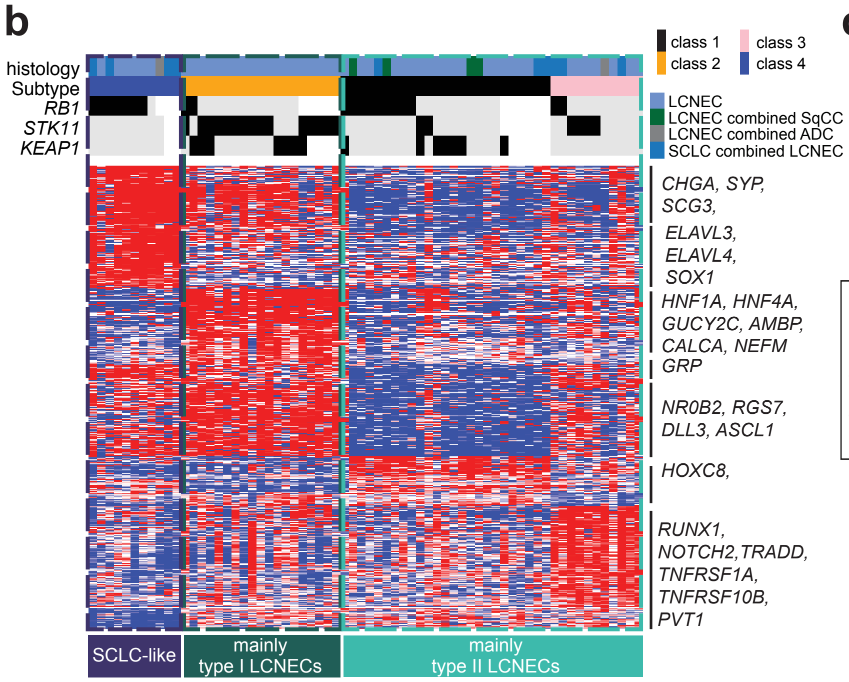

c

LCNEC - SCLC cluster (Fig.3, Suppl. Fig. 11)

LCNEC cluster		mainly type I LCNECs: class I	mainly type II LCNECs: class II	mainly SCLC: class III	mainly SCLC: class IV
SCLC-like	class 4	0	1	3	7
mainly type I LCNECs	class 2	18	1	0	0
mainly type II LCNECs	class 1	1	24	0	0
mainly type II LCNECs	class 3	0	10	1	0

$P < 0.001$

Supplementary Fig. 13: Expression clustering of LCNECs. (a) Consensus clustering was applied to 66 LCNECs and the matrix for the cluster solutions k=3, k=4 and k=5 is provided in the top panel. PCA and silhouette plots for all cluster solutions are provided in the middle and bottom panel, respectively. Solution k=4 was chosen for subsequent studies (red box). (b) The clustering results for LCNECs are displayed as a heatmap referring to ClaNC rank genes (Methods section); tumor samples are arranged in columns, annotated for the histological subtype, for the transcriptional classes 1-4, and for the somatic alteration status according to the color panel. Selected candidate genes are shown. Dashed lines highlight the assignments as identified in Figure 3a. (c) Contingency table for clustering results based on the LCNEC cluster (classes 1-4) and the LCNEC-SCLC cluster (classes I-IV, Figure 3). Red boxes indicate the assignments defined in Figure 3. Statistical significance was determined by Fisher's exact test, Monte Carlo version (10000 permutations, P<0.001).

Supplementary Figure 14

LCNEC and SCLC tumors: stage I and II (n=42)

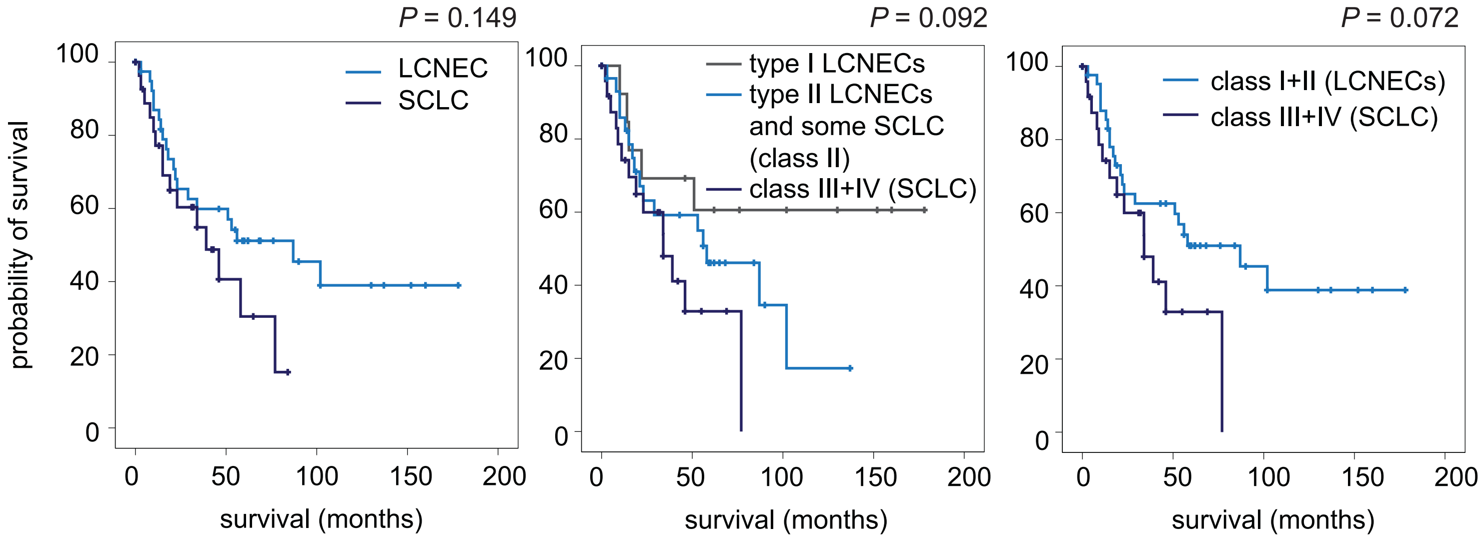

Supplementary Fig. 14: Clinical correlations of transcriptional subsets defined for LCNEC and SCLC patients. The overall survival was determined for LCNEC and SCLC patient tumors diagnosed at stage I and II. Subcategories were defined by the histological annotation (left panel), by transcriptional subgroups formed either by type I LCNECs, type II LCNECs and some SCLC tumors (transcriptional class II) or SCLC tumors of transcriptional class III and IV (middle panel). Furthermore, transcriptional classes I and II (mainly containing LCNECs) were compared to the transcriptional classes III and IV (mainly SCLC) at the right panel. Statistical significance values were calculated by log-rank test ($P<0.05$).

Supplementary Table 1. Antibodies for immunohistochemistry

CD56 (*NCAM1*)	Cell Marque (Rocalin, California, USA),<br>156R-95, clone: MRQ-42	1/200
Synaptophysin (*SYP*)	Cell Marque (Rocalin, California, USA),<br>336R-96, clone: MRQ-40	1/100
Chromogranin A (*CHGA*)	Chemicon/Fisher Scientific (Hampton, New Hampshire, USA),<br>MAB5268, clone: LK2H10	1/1000
TTF1 (*NKX2.1*)	NeoMarkers (Portsmouth, New Hampshire, USA)<br>MS-699-P, clone 8G7G3/1	1/300
Rb1 (*RB1*)	Abcam (Cambridge, UK), ab81701, clone 1F8	1μg/mL
