# Supplementary material for: Integrative genomic profiling of large-cell neuroendocrine carcinomas reveals distinct subtypes of high-grade neuroendocrine lung tumors
Source: Nat Commun. 2018 Mar 13;9:1048. doi: 10.1038/s41467-018-03099-x (PMC5849599; doi:10.1038/s41467-018-03099-x)
Supplement: Supplementary file 2 — Description of Additional Supplementary Files [file 41467_2018_3099_MOESM2_ESM.pdf]

Description of Supplementary Files

File Name: Supplementary Data 1
Description: Sample overview

File Name: Supplementary Data 2
Description: Histological evaluation on LCNECs

File Name: Supplementary Data 3
Description: Mutation signature analysis

File Name: Supplementary Data 4
Description: Significant copy number alterations

File Name: Supplementary Data 5
Description: Samples with copy number alterations

File Name: Supplementary Data 6
Description: Mutation calls

File Name: Supplementary Data 7
Description: Significantly mutated genes

File Name: Supplementary Data 8
Description: Allelic states for TP53, RB1, STK11 and KEAP1

File Name: Supplementary Data 9
Description: Genomic rearrangements

File Name: Supplementary Data 10
Description: Chimeric transcripts

File Name: Supplementary Data 11
Description: Expression data of LCNECs

File Name: Supplementary Data 12
Description: Lung cancer subtypes: annotation for clustering class and somatic alterations

File Name: Supplementary Data 13
Description: ClaNC classifier genes and differentially expressed genes

File Name: Supplementary Data 14
Description: DAVID analysis
